# Fam172a Mediates the Stimulation of Hypothalamic Oxytocin Neurons to Suppress Obesity‐Induced Anxiety

**DOI:** 10.1002/advs.202414723

**Published:** 2025-02-17

**Authors:** Baocheng Wan, Lina Zhang, Xinyu Wang, Rong Zhang, Lianxi Li, Yi Zhang, Zhuo Chen, Cheng Hu

**Affiliations:** ^1^ Jinzhou Medical University Graduate Training Base Shanghai Sixth People's Hospital Shanghai Jiao Tong University School of Medicine Shanghai 200233 China; ^2^ School of Public Health Shanghai Jiao Tong University School of Medicine Shanghai 200025 China; ^3^ School of Life Science and Technology ShanghaiTech University Shanghai 201210 China; ^4^ Shanghai Diabetes Institute Shanghai Key Laboratory of Diabetes Mellitus Shanghai Clinical Center for Diabetes Shanghai Sixth People's Hospital Affiliated to Shanghai Jiao Tong University School of Medicine Shanghai 200233 China; ^5^ Department of Endocrinology and Metabolism Fengxian Central Hospital Affiliated to Southern Medical University Shanghai 201449 China

**Keywords:** ago2, anxiety, fam172a, obesity, oxytocin neurons

## Abstract

Anxiety disorder is the most common mental disorder worldwide. Although human studies have demonstrated a positive association between obesity and anxiety disorder, the exact mechanism linking these conditions is unclear. Interestingly, oxytocin (Oxt) neurons, predominantly expressed in the hypothalamic paraventricular nucleus (PVN), play a crucial role in both obesity and anxiety. In this study, obesity can induce anxiety‐like behavior in mice, which can be ameliorated by the activation of PVN Oxt neurons. Conversely, inhibiting PVN Oxt neurons accelerate the progression of anxiety. Moreover, the family with sequence similarity 172, member A (Fam172a), an anxiety susceptibility gene, is highly expressed in the hypothalamic PVN Oxt neuron but reduce in the PVN Oxt neuron of mice in the high‐fat diet and acute restraint stress conditions. Significantly, overexpression of Fam172a in PVN Oxt neurons improve obesity‐anxiety‐like behavior in mice. In contrast, disruption of Fam172a in PVN Oxt neurons exacerbate obesity‐anxiety‐like behavior. Furthermore, this study demonstrates that Fam172a is involved in mRNA degradation in Oxt neurons by regulating the intranuclear transport of Argonaute 2, thereby influencing Oxt secretion and ultimately impacting obesity‐anxiety‐like behavior. These findings suggest that Fam172a serves as a key target of PVN Oxt neurons in the regulation of obesity‐induced anxiety.

## Graphical abstract

This study specifically focuses on the role of oxytocin neurons in the hypothalamic paraventricular nucleus (PVN) in regulating obesity‐induced anxiety in mouse models. By combining chemogenetic, genetic, and pharmacological approaches, it is systematically demonstrated that oxytocin neurons in the PVN regulate anxiety induced by obesity through the Fam172a gene.

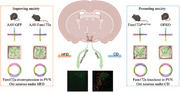

## 1 Introduction

Anxiety is a common emotional response, and anxiety disorders often arise from irrational fears and worries related to anticipated events, objects, or time periods, resulting in a sense of apprehension and dread, which affects an individual's thoughts, feelings, abilities, and behavior.^[^ ^1^ ^]^ The prevalence of anxiety disorders is consistently increasing and is one of the largest contributors to non‐fatal health losses.^[^ ^2^ ^]^ Obesity can significantly influence mood regulation and mental health,^[^ ^3^ ^]^ contributing to the occurrence and development of anxiety. Studies have indicated that diet‐induced obesity (DIO) can disrupt brain function, leading to an increased prevalence of anxiety disorders.^[^ ^4^ ^]^ Meanwhile, malfunctioning of the central nervous system (CNS) has been noted as a primary factor contributing to the development of anxiety disorders.^[^ ^5^ ^]^ The hypothalamus, as a higher center of the CNS, plays a crucial role in regulating the production and expression of emotions.^[^ ^6^ ^]^ However, the association between anxiety, obesity, and the CNS is unclear.

Previous studies have reported that the intraperitoneal administration of 1 mg kg^−1^ oxytocin (Oxt) in mice can reverse the functional brain impairments induced by DIO.^[^ ^7^ ^]^ Oxt neurons act as a nonapeptide hormone, mainly synthesized in the paraventricular nucleus (PVN) of the hypothalamus. During anxiety states, Oxt neurons are activated and Oxt is released into the blood to exert an anti‐anxiety effect.^[^ ^8^ ^]^ Epidemiological research has shown a significant inverse relationship between anxiety levels and serum Oxt concentrations.^[^ ^9^ ^]^ Similarly, mice genetically deficient in Oxt exhibited behaviors indicative of anxiety.^[^ ^10^ ^]^ Furthermore, multiple studies have demonstrated the effectiveness of Oxt treatment in relieving anxiety^[^ ^11^ ^]^ and DIO.^[^ ^12^ ^]^ Thus, Oxt neurons play a crucial role in anxiety regulation and hold promise as a potential treatment for anxiety disorders.^[^ ^13^ ^]^ However, the precise mechanism underlying the relationship between obesity‐induced anxiety and Oxt remains unclear.

A study involving whole blood genomic testing of Han Chinese patients with generalized anxiety disorder and obsessive‐compulsive disorder^[^ ^14^ ^]^ reported a series of genes with susceptibility to anxiety, among which family with sequence similarity 172, member A  (Fam172a) showed high expression in the hypothalamus, especially the PVN Oxt neurons. In this study, we found a reduction in the expression of the Fam172a gene within PVN Oxt neurons in high‐fat diet (HFD)‐induced anxiety model mice. Fam172a is a novel protein identified by the research team from aortic tissue.^[^ ^15^ ^]^ Recent studies have identified Fam172a as a participant in the pathophysiology of multiple diseases, including thyroid cancer,^[^ ^16^ ^]^ colorectal cancer,^[^ ^17^ ^]^ pancreatic cancer,^[^ ^18^ ^]^ osteosarcoma,^[^ ^19^ ^]^ CHARGE syndrome,^[^ ^20^ ^]^ and atherosclerosis.^[^ ^21^ ^]^ Fam172a directly regulates the function of the Argonaute 2 (Ago2) gene by facilitating the nuclear movement of Ago2.^[^ ^20^ ^]^ Therefore, the expression of Fam172a in PVN Oxt neurons may play a significant role in anxiety induced by obesity.

In this study, we focused on the specific role of PVN Oxt neurons in regulating obesity‐induced anxiety. By combining chemogenetic, genetic, and pharmacological approaches, we systematically demonstrate that Oxt neurons in the PVN regulate anxiety induced by obesity through Fam172a. Furthermore, this study clarifies the specific mechanisms involved, providing novel insights for the treatment of obesity‐related anxiety disorders.

## 2 Results

### 2.1 HFD Promotes the Progression of Anxiety Under Stress

Obesity which contributes to the occurrence and development of anxiety results from a complex interplay between genetic and environmental causes, in which diet is the main influencing factor.^[^ ^22^ ^]^ However, the role of a HFD in the progression of anxiety is unclear. To explore this problem, we introduce a classical anxiety model, the chronic restraint stress (CRS) anxiety model.^[^ ^23^ ^]^ First, we did a 7‐day chronic restraint stress experiment, and the results consistently proved the reliability of the classical model (Figure S1A–Q, Supporting Information). We then performed an acute three‐day restraint stress (ARS) experiment based on this model and found that it did not cause anxiety‐like performance in male mice (Figure S2A–L, Supporting Information). Further, we introduced mice fed a chow diet (CD) or a high‐fat diet (HFD) on the basis of normal or ARS experiment (Figure S2N, Supporting Information), and found that mice with ARS‐HFD displayed shorter time (Figure S2B, Supporting Information) and less frequent times (Figure S2C, Supporting Information) in the open arm in elevated plus‐maze (EPM) test. Additionally, in the open field test (OFT) and elevated zero maze test (EZM) (Figure S2E–I, Supporting Information), ARS‐HFD mice also displayed heightened anxiety levels which presented shorter time in the center (Figure S2F, Supporting Information) and open arms (Figure S2J, Supporting Information). Notably, there were no differences among groups in terms of exploration frequency and total distance traveled during any of these tests (Figure S2D, G, H, K, and L, Supporting Information). To eliminate potential sex differences, we administered identical treatments and behavioral assessments (Figure S3A–L, Supporting Information) to female mice. The results exhibited consistency across the sexes. Thus, under conditions of stress, HFD may facilitate the development of anxiety.

### 2.2 Mice Exhibit Anxiety‐Like Behaviors After Long‐Term Consumption of HFD

**Figure 1:**
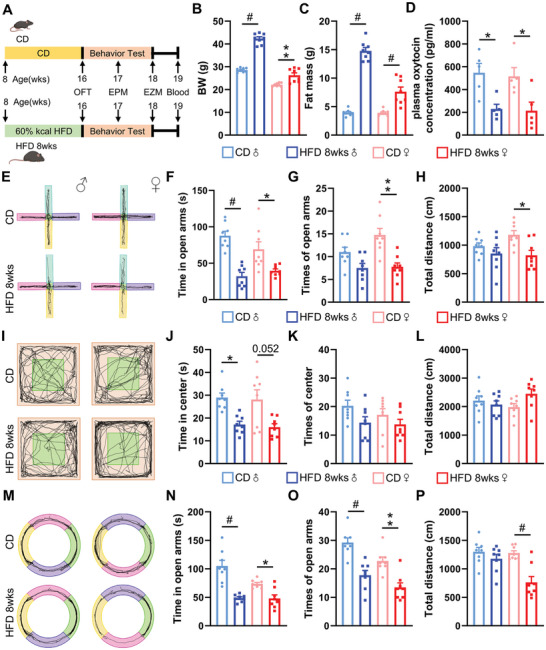
A HFD induces anxiety‐like behaviors in mice. A) 8‐week‐old mice were randomly assigned to two groups and fed either a CD or HFD until they attained 16 weeks of age. Subsequently, behavioral assessments were performed, accompanied by measurements of body weight, analyses of body composition, and extraction of tissue samples. Created in BioRender. Chen, Z. (2024) https://BioRender.com/m15b213. B,C) Following the assessment of anxiety‐like behaviors, measurements of body weight (B) and body composition (C) were conducted. *n* = 8 (CD, male), 8 (HFD 8wks, male), 8 (CD, female), or 8 (HFD 8wks, female) mice per group. D) Plasma was extracted from CD or HFD 8wks mice, and plasma Oxt levels were determined. *n* = 5 for each group. E–H) Anxiety‐like behavior was assessed in CD or HFD 8wks mice. The activity trajectory line plot (E), time spent in open arms (F), times of open arms (G), and total distance (H) of CD or HFD 8wks mice were assessed in the EPM. *n* = 8 (CD, male), 8 (HFD 8wks, male), 8 (CD, female), or 8 (HFD 8wks, female) mice per group. I–L) The mice activity trajectory line plot (I), time in center (J), times of center (K), and total distance (L) were assessed in the OFT. *n* = 8 (CD, male), 8 (HFD 8wks, male), 8 (CD, female), or 8 (HFD 8wks, female) mice per group. M–P) The mice activity trajectory line plot (M), time in open arms (N), times of open arms (O), and total distance (P) were assessed in the EZM. *n* = 8 (CD, male), 8 (HFD 8wks, male), 8 (CD, female), or 8 (HFD 8wks, female) mice per group. The data in (B) to (D), (F) to (H), (J), and (N) to (P) are presented as mean ± SEM. ^*^ *p* < 0.05, ^**^ *p* < 0.01, #*p* < 0.001, *p*‐values are calculated using two‐way ANOVA with Tukey correction (B–D, F–H, J–L, N–P).

In most cases, obesity is usually caused by a long‐term HFD.^[^ ^3b^ ^]^ Therefore, in the long‐term HFD conditions, whether it can cause anxiety in mice and its related mechanism is what we will explore next. To simulate an obese state, eight‐week‐old *C57BL/6* mice were subjected to an eight‐week HFD (HFD 8wks) regimen (**Figure** **1**A). Both male and female mice in the HFD 8wks group exhibited increases in body weight (Figure 1B) and fat mass (Figure 1C). We then performed behavioral experiments on these diet‐induced obese mice and control mice. In the EPM (Figure 1E), irrespective of sex, the HFD 8wks group spent less time in the open arms (Figure 1F), and female HFD 8wks mice did less frequency entries into the open arms (Figure 1G) and shorter distance traveled (Figure 1H). Meanwhile, in the OFT (Figure 1I) and EZM (Figure 1M), both male and female HFD 8wks mice spent less time in the central zone and open arms (Figure 1J,N). During the OFT, there were no variations in either the frequency of exploration or the total distance traveled (Figure 1K,L). However, in the EZM, HFD 8wks mice had fewer entries into the open arms, regardless of sex (Figure 1O), and HFD 8wks female mice traveled a shorter distance (Figure 1P). These results showed that the long‐term HFD mice exhibited increased anxiety‐like behaviors.

There are numerous studies have shown that the PVN Oxt neurons in the hypothalamus and its released Oxt are closely related to anxiety‐like behavior.^[^ ^7^, ^24^ ^]^ Thus, plasma samples of the experiments mentioned above were collected to measure Oxt levels. Irrespective of sex, ARS‐CD mice exhibited an increase in plasma Oxt levels, whereas ARS‐HFD showed a decreased level of Oxt (Figures S2M and S3M, Supporting Information). And HFD 8wks mice also presented a decrease in plasma Oxt levels (Figure 1D). In the presence of altered Oxt release, we also examined the activity of PVN Oxt neurons in the hypothalamus. Brain slices were prepared from *C57BL/6* mice exposed to either CD, ARS, or CRS, and c‐Fos was used to demonstrate neuronal activity. Enhanced PVN Oxt neuronal activity was observed in ARS mice, whereas a decrease in Oxt neuronal activity was noted in the PVN of CRS mice (Figure S4A–D, Supporting Information). Additionally, brain slices from eight‐week‐old *Oxt^Cre^ Rosa* mice that were subjected to either CD, ARS‐CD, ARS‐HFD or HFD 8wks, and performed immunofluorescence staining. The data revealed that ARS‐HFD mice exhibited a decrease in the number of c‐Fos cells within PVN Oxt neurons (Figure S4C,D, Supporting Information). And HFD 8wks mice also showed a decreased activity of PVN Oxt neurons (Figure S4E,F, Supporting Information). These data suggested that PVN Oxt neurons and their released Oxt were closely related to anxiety. In addition, under stress conditions or obesity conditions, HFD could inhibit the activity of PVN Oxt neurons and reduce Oxt release.

### 2.3 PVN Oxt Neurons Play a Significant Role in Regulating HFD‐Induced Anxiety

**Figure 2:**
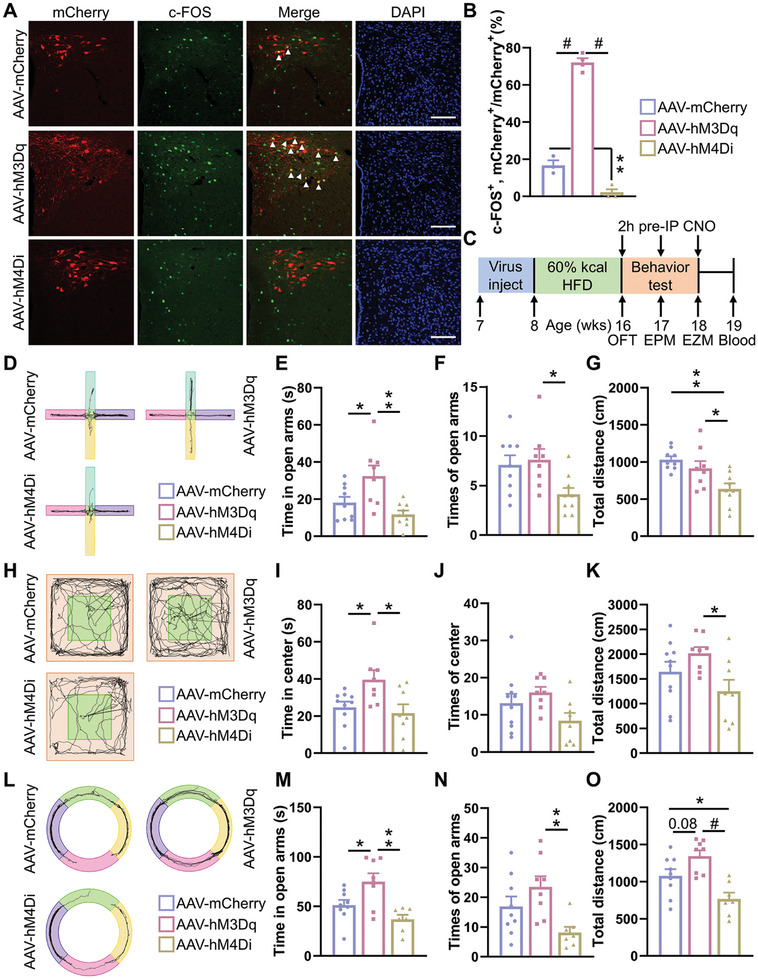
PVN Oxt neurons are involved in regulating anxiety‐like behavior. A) Expression of mCherry (red) after bilateral injection of AAV‐hSyn‐DIO‐mCherry (AAV‐mCherry), AAV‐hSyn‐DIO‐hM3Dq‐mCherry (AAV‐hM3Dq), and AAV‐hSyn‐DIO‐hM4Di‐mCherry (AAV‐hM4Di) viruses into the PVN of *Oxt^Cre^* mice. Brain sections of the mice were immunostained for c‐Fos (green), a neuronal activity marker. The section was counter‐stained with DAPI (blue). Arrowheads indicate cells co‐expressed by mCherry and c‐Fos. Scale bars, 100 µm. B) Percentage of mCherry^+^ cells expressing c‐Fos in PVN Oxt cells of mice. *n* = 3 (AAV‐mCherry), 4 (AAV‐hM3Dq), or 3 (AAV‐hM4Di) mice per group. C) Schematic representation of the timeline for anxiety‐like behavioral planning in mice. The intraperitoneal injection (IP) was performed 2 h prior to the desired experimental procedure (2h pre‐IP CNO). D–G) eight‐week‐old male *Oxt^Cre^* mice were injected with Cre inducible AAV‐mCherry, AAV‐hM3Dq, and AAV‐hM4Di viruses into the PVN and were placed on a HFD for eight weeks. The activity trajectory line plot (D), time in open arms (E), times of open arms (F), and total distance (G) of AAV‐mCherry, AAV‐hM3Dq, and AAV‐hM4Di mice were assessed in the EPM. *n* = 9 (AAV‐mCherry), 8 (AAV‐hM3Dq), or 9 (AAV‐hM4Di) mice per group. H–K) The mice activity trajectory line plot (H), time in center (I), times of center (J), and total distance. (K) were assessed in the OFT. *n* = 10 (AAV‐mCherry), 8 (AAV‐hM3Dq), or 8 (AAV‐hM4Di) mice per group. L–O) The mice activity trajectory line plot (L), time in open arms (M), times of open arms (N), and total distance (O) were assessed in the EZM. *n* = 9 (AAV‐mCherry), 8 (AAV‐hM3Dq), or 7 (AAV‐hM4Di) mice per group.The data in (B), (E) to (G), (I), (K), and (M) to (O) are presented as mean ± SEM.^*^ *p* < 0.05, ^**^ *p* < 0.01, #*p* < 0.001, *p*‐values are calculated using one‐way ANOVA with Tukey correction (B, E‐G, I‐K, M‐O).

To determine whether PVN Oxt neurons modulate anxiety associated with HFD, we used designer receptors exclusively activated by designer drugs and their ligand clozapine N‐oxide (CNO) to manipulate Oxt neuronal activity in the PVN.^[^ ^25^ ^]^ We injected Cre‐dependent adeno‐associated virus (AAV)‐hSyn‐DIO‐mCherry, AAV‐hSyn‐DIO‐hM3Dq‐mCherry, and AAV‐hSyn‐DIO‐hM4Di‐mCherry into the bilateral PVN of *Oxt^Cre^* mice, resulting in mCherry expression specifically in PVN Oxt neurons. In *Oxt^Cre^* mice expressing hM3Dq‐mCherry (activation group), CNO injection increased c‐Fos positive cells in the PVN after 2 h, indicating neuron activation. Conversely, in mice expressing hm4di‐mCherry (inhibition group), the same treatment decreased the activity of these cells, indicating neuron inhibition (**Figure** **2**A,B).

We also controlled the activity of PVN Oxt neurons in mice under different models to observe whether it affected the anxiety‐like behavior of mice. First, in CD, ARS‐CD, and ARS‐HFD groups, mice were injected with each of the three viruses described above and underwent behavioral experiments after recovery. In the OFT (Figure S5A, Supporting Information), EPM (Figure S5K, Supporting Information) and EZM (Figure S5U, Supporting Information), the inhibition of PVN Oxt neurons showed more anxiety symptoms in the CD or ARS‐CD group which showed normal behavior before virus treatment (Figure S5B–G, L–Q, V–AA, Supporting Information). However, in ARS‐HFD group which showed anxiety‐like behavior, the activation of PVN Oxt neurons could relieve anxiety‐like behavior (Figure S5H–J, R–T, AB–AD, Supporting Information). Then, in the long‐term HFD group, mice were also injected with each of the three viruses and underwent behavioral experiments after recovery (Figure 2C). In the EPM (Figure 2D–G), OFT (Figure 2H–K), and EZM (Figure 2L–O), the mice exhibited anxiety‐like behavior as a result of being fed a HFD for a long time, therefore, only the PVN Oxt neuron‐activated group showed an improvement in anxiety‐like behavior such as spent more time in the open arms or center. There was no obvious difference between the control group and the PVN Oxt neuron‐inhibited group. In addition, the PVN Oxt neuron‐inhibited group showed reduced overall activity. These results suggested that it was possible to influence anxiety‐like behavior in mice by regulating the activity of PVN Oxt neurons.

To mitigate the potential impact of sex differences on experimental outcomes, we exposed female mice to the same treatments as their male counterparts. The results of anxiety‐like behavioral assessments revealed comparable behavioral responses between male and female mice, both in studies involving CD, ARS‐CD, and ARS‐HFD female mice (Figure S6A–AD, Supporting Information), as well as in those focusing on eight‐week‐old CD and HFD female mice (Figure S7A–L, Supporting Information). These results showed that activating PVN Oxt neurons in both male and female mice reduced HFD‐induced anxiety‐like behaviors, while inhibition exacerbated it. Overall, PVN Oxt neurons were crucial for regulating such behaviors.

### 2.4 Down‐Regulation of Fam172a Expression in the PVN Oxt Neurons of HFD‐Fed Mice

**Figure 3:**
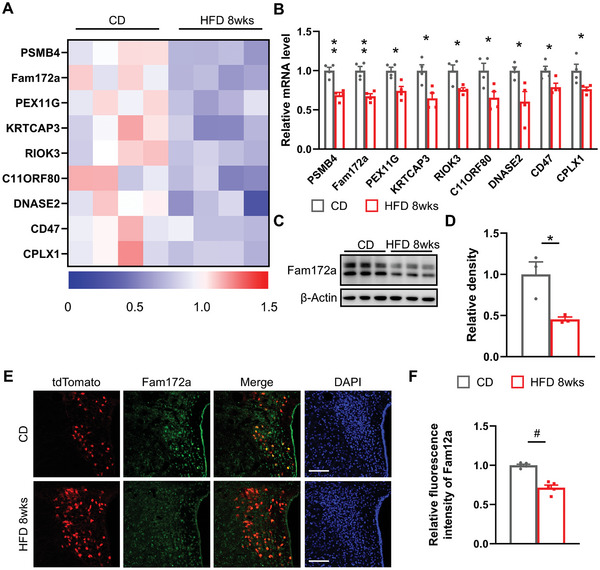
Fam172a expression is decreased in Oxt neurons of eight‐week HFD‐fed mice. A,B) Total RNA was extracted from the hypothalamus of mice treated with CD and HFD 8wks, and the mRNA expression of differential genes was shown in heatmaps (A) and histograms (B). *n* = 4 samples per group. C,D) The results of Western blot of the hypothalamus in CD mice and HFD 8wks mice (C), and specific results and relative intensity analyses (D). β‐actin was used as a loading control. *n* = 3 samples per group. E) Immunofluorescence staining of Fam172a (green) was performed on brain sections of CD *Oxt^Cre^ Rosa* mice and HFD 8wks *Oxt^Cre^ Rosa* mice. Cell nuclei were counter‐stained with DAPI (blue). Scale bars, 100 µm. F) Relative intensity of Oxt cells expressing Fam172a in the PVN of CD mice and HFD 8wks mice. *n* = 5 (CD) or 5 (HFD 8wks) mice per group.The data in (B), (D), and (F) are presented as mean ± SEM.^*^ *p* < 0.05, ^**^ *p* < 0.01, #*p* < 0.001, and *p*‐values are calculated using two‐tailed Student's *t*‐test (B, D, F).

Based on previous research that investigated DNA methylation in whole blood cell sequencing in Chinese Han patients with anxiety disorders,^[^ ^14^ ^]^ we selected 28 differential genes and further investigated whether these genes are involved in HFD‐induced anxiety by impacting Oxt neurons. Then, we verified the mRNA expression levels of these genes in the hypothalamus of both the CD and the HFD 8wks groups. Eventually, we focused on Fam172a due to its observed reduction in expression within the HFD 8wks groups (**Figure** **3**A,B). Similarly, Western blot analysis revealed decreased levels of Fam172a protein in the hypothalamus of the HFD 8wks groups (Figure 3C,D). To examine Fam172a expression in PVN Oxt neurons, we performed immunofluorescence staining on brain slices from CD and HFD 8wks *Oxt^Cre^ Rosa* mice. Results showed reduced Fam172a fluorescence intensity in hypothalamic PVN Oxt neurons of the HFD 8wks group (Figure 3E,F). In summary, Fam172a may play a role in modulating HFD‐induced anxiety‐like behavior through Oxt neurons located in the PVN.

### 2.5 Overexpression of Fam172a in PVN Oxt Neurons Protects Against HFD‐Induced Anxiety

**Figure 4:**
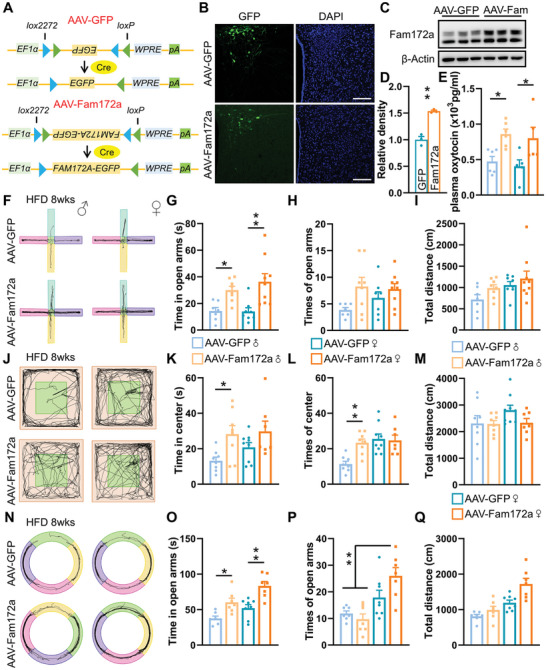
Overexpression of Fam172a in Oxt neurons inhibits the progression of HFD‐induced anxiety. A)Schematic representation of AAV‐GFP and AAV‐Fam172a constructed viruses. B) After injecting AAV‐GFP and AAV‐Fam172a into the PVN of adult *Oxt^Cre^* mice, which could lead to EGFP expression (green). Cell nuclei were counter‐stained with DAPI (blue). Scale bars, 100 µm. C,D) Mouse hypothalamus was injected with AAV‐GFP and AAV‐Fam172a and hypothalamic lysates were extracted for Western blot analysis of Fam172a (C) and quantification of the Western blots for AAV‐GFP and AAV‐Fam172a (D). *n* = 3 for each group. E) AAV‐GFP or AAV‐Fam172a viruses were injected into the hypothalamic PVN of *Oxt^cre^* mice. At week 19 of the HFD, plasma samples were collected, and Oxt levels were measured. *n* = 6 (AAV‐GFP, male), 6 (AAV‐Fam172a, male), 5 (AAV‐GFP, female), or 5 (AAV‐Fam172a, female) mice per group. F–I) eight‐week‐old male and female *Oxt^Cre^* mice were injected with Cre inducible AAV‐GFP and AAV‐Fam172a viruses into the PVN and fed a HFD for eight weeks. The level of anxiety in the mice was assessed. The activity trajectory line plot (F), time in open arms (G), times of open arms (H), and total distance (I) of the AAV‐GFP and AAV‐Fam172a mice were assessed in the EPM. *n* = 7 (AAV‐GFP, male), 8 (AAV‐Fam172a, male), 8 (AAV‐GFP, female), or 9 (AAV‐Fam172a, female) mice per group. J–M) The mice activity trajectory line plot (J), time in center (K), times of center (L), and total distance (M) were assessed in the OFT. *n* = 8 (AAV‐GFP, male), 8 (AAV‐Fam172a, male), 9 (AAV‐GFP, female), or 7 (AAV‐Fam172a, female) mice per group. N–Q) The mice activity trajectory line plot (N), time in open arms (O), times of open arms (P), and total distance (Q) were assessed in the EZM. *n* = 7 (AAV‐GFP, male), 7 (AAV‐Fam172a, male), 8 (AAV‐GFP, female), or 7 (AAV‐Fam172a, female) mice per group.The data in (D), (E), (G), (K), (L), (O), and (P) are presented as mean ± SEM.^*^ *p* < 0.05; ^**^ *p* < 0.01, *p*‐values are calculated using two‐tailed Student's *t*‐test (D), two‐way ANOVA with Tukey correction (E, G‐I, K‐M, O‐Q).

To further investigate the function of Fam172a in Oxt neurons, we developed an AAV plasmid designed to ensure sustained expression of Fam172a specifically in the presence of Cre recombinase (**Figure** **4**A). Both AAV‐GFP and AAV‐Fam172a viruses were injected into the PVN of *Oxt^Cre^* mice, with immunofluorescence confirming the precise localization of the viruses (Figure 4B). Western blot analysis demonstrated overexpression of Fam172a in *Oxt^Cre^* mice, indicating the effectiveness of the viruses (Figure 4C,D). After surgery, control and overexpressing mice were split into pairs, one group on a CD, and the other group on a HFD. Our findings showed both male and female mice gained weight and fat mass under HFD condition (Figure S8A,B, Supporting Information). Under HFD conditions, both male and female mice in the AAV‐Fam172a group exhibited higher plasma Oxt concentrations (Figure 4E). Under HFD conditions, in the EPM (Figure 4F), Fam172a‐overexpressing mice spent more time in the open arms but showed no change in the number of entries (Figure 4G,H). In the OFT (Figure 4J), male mice overexpressing Fam172a stayed longer and entered the center zone more frequently, whereas no significant differences were observed between female groups (Figure 4K,L). In the EZM (Figure 4N), overexpressing Fam172a mice explored the open arms for longer durations (Figure 4O), especially females which did more frequent entries into the open arms than males (Figure 4P). There were no significant differences in total distance traveled were noted among the groups (Figure 4I,M,Q). However, in the OFT (Figure S8C–F, Supporting Information), EPM (Figure S8G–J, Supporting Information), and EZM (Figure S8K–N, Supporting Information), there were no statistically differences in anxiety‐related behaviors were observed under CD conditions. Based on these results, we concluded that overexpressing Fam172a in Oxt neurons effectively alleviates anxiety symptoms caused by obesity in both male and female mice.

### 2.6 Deleting Fam172a in Oxt Neurons Induces Anxiety‐Like Behaviors in Mice

**Figure 5:**
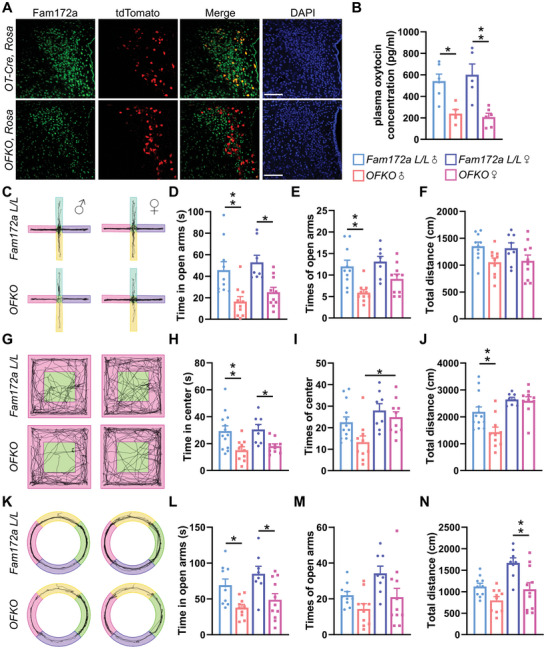
Deleting Fam172a in Oxt neuronal cells induces anxiety‐like behavior in standard chow‐fed mice. A) Immunofluorescence indicated that Fam172a (green) in Oxt neurons of the *Oxt^Cre^, Fam172a L/L* (*OFKO*) mice was depleted. Oxt neurons in *Oxt^Cre^ Rosa* mice and *OFKO Rosa* mice could be expressed as tdTomato (red) cells. Scale bars, 100 µm. B) Plasma was extracted from *Fam172a L/L* mice and *OFKO* mice under the chow diet, and serum Oxt levels were determined. *n* = 6 (*Fam172a L/L*, male), 5 (*OFKO*, male), 5 (*Fam172a L/L*, female), or 6 (*OFKO*, female) mice per group. C–F) Anxiety‐like behavior was measured in *Fam172a L/L* and *OFKO* mice on a normal chow diet. The activity trajectory line plot (C), time in open arms (D), times of open arms (E), and total distance (F) of *Fam172a L/L* and *OFKO* mice were assessed in the EPM. *n* = 10 (*Fam172a L/L*, male), 10 (*OFKO*, male), 8 (*Fam172a L/L*, female), or 10 (*OFKO*, female) mice per group. G–J) The mice activity trajectory line plot (G), time in center (H), times of center (I), and total distance (J) were assessed in the OFT. *n* = 13 (*Fam172a L/L*, male), 10 (*OFKO*, male), 8 (*Fam172a L/L*, female), or 10 (*OFKO*, female) mice per group. K–N) The mice activity trajectory line plot (K), time in open arms (L), times of open arms (M), and total distance (N) were assessed in the EZM. *n* = 10 (*Fam172a L/L*, male), 10 (*OFKO*, male), 8 (*Fam172a L/L*, female), or 11 (*OFKO*, female) mice per group.The data in (B), (D), (E), (H) to (J), (L), and (N) are presented as mean ± SEM. ^*^ *p* < 0.05, ^**^ *p* < 0.01, *p*‐values are calculated using two‐way ANOVA with Tukey correction (B, D‐F, H‐J, L‐N).

Next, we generated *Oxt^Cre^ Fam172a^loxP/loxP^* (*OFKO*) mice by crossing *Fam172a^loxP/loxP^* (*Fam172a L/L*) with *Oxt^Cre^* mice. Meanwhile, littermate *Fam172a L/L* mice were used as controls. Immunofluorescence staining confirmed the absence of Fam172a in Oxt neurons in *OFKO* mice (**Figure** **5**A). Additionally, we observed reduced Oxt plasma levels in *OFKO* mice (Figure 5B), suggesting a close relationship between Fam172a and Oxt secretion in Oxt neurons. To ensure experimental consistency, 16‐week‐old mice were tested. In the EPM (Figure 5C), OFT (Figure 5G), and EZM (Figure 5K), *OFKO* mice displayed anxiety‐like behaviors by spending more time in open arms and central zone (Figure 5D,H,L) in both male and female mice. *OFKO* male mice made fewer the number of entries in open arms and central zones (Figure 5E,I,M), and *OFKO* mice exhibited less total movement (Figure 5F,J,N). In summary, knockout of Fam172a in Oxt neurons resulted in decreased Oxt release, and induced anxiety‐like behaviors in mice.

### 2.7 Fam172a Modulates Anxiety Symptoms by Influencing the Stability of the Oxt Neuron mRNA

**Figure 6:**
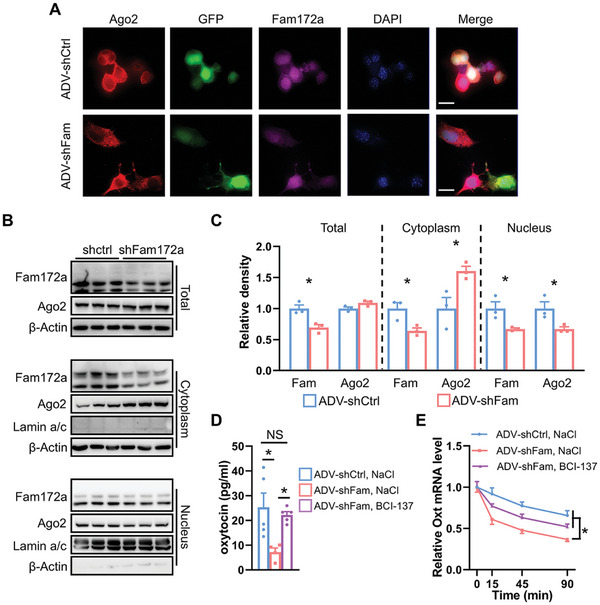
Fam172a regulates mRNA degradation by mediating Ago2 entry into the cytoplasm. A) The Neuro2a neuronal cells were transfected with vehicle ADV‐shCtrl or ADV‐shFam172a adenovirus at 37 °C for 48 h. Then, the co‐localization of GFP, Fam172a, and Ago2 in Neuro2a cells was detected by immunofluorescence staining. Scale bars, 20 µm. B,C) Neuro2a cells were incubated with ADV‐shCtrl (shCtrl) or ADV‐shFam172a (shFam172a) at 37 °C for 48 h. Total proteins and cytoplasmic and nuclear proteins were extracted; cytoplasmic and nuclear proteins used NE‐PER extraction reagents. Western blots of Fam172a and Ago2 were shown (B), and densitometry of the total proteins, cytoplasmic, and nuclear proteins blots is shown (C). *n* = 3 cell cultures per group. D) After 24 h post‐treatment of the Neuro2a cells with ADV‐shCtrl + NaCl, ADV‐shFam172a + NaCl or ADV‐shFam172a + BCI‐137 (100 nM), Oxt levels of media were determined. *n* = 5 (ADV‐shCtrl, NaCl), 4 (ADV‐shFam172a, NaCl), or 5 (ADV‐shFam172a, BCI‐137) samples per group. E) After treatment of Neuro2a cells with ADV‐shCtrl + NaCl, ADV‐shFam172a + NaCl or ADV‐shFam172a + BCI‐137, decay of the Oxt gene of Neuro2a cells was shown using actinomycin D (4 µg mL^−1^). The data in (C) to (E) are presented as mean ± SEM. ^*^ *p* < 0.05, *p*‐values are calculated using two‐tailed Student's *t*‐test (C), one‐way ANOVA with Tukey correction (D), two‐way ANOVA with Bonferroni correction (E).

Previous reports suggest that Fam172a interacts with Ago2 and is involved in transcriptional regulation.^[^ ^26^ ^]^ In our study, Neuro2a cells were transfected with either adenovirus (ADV) ‐shRNA‐EGFP (ADV‐shCtrl) or ADV‐shFam172a‐EGFP (ADV‐shFam172a) for a duration of 48 h. Immunofluorescence staining of Fam172a knockdown Neuro2a cells revealed an increased expression of Ago2 in the cytoplasm (**Figure** **6**A). Protein analysis confirmed that Fam172a expression decreased, while Ago2 protein levels remained relatively constant after ADV‐shFam172a treatment. Furthermore, we observed a decrease in Fam172a expression in both the nucleus and cytoplasm, along with a reduction in nuclear translocation of Ago2 in shFam172a cells (Figure 6B,C). Next, we found that the Oxt concentration in the medium solution of Neuro2a cells transfected with ADV‐shFam172a and treated with NaCl was reduced, however, the Oxt concentration in the medium solution of Neuro2a cells transfected with ADV‐shFam172a and treated with BCI‐137,^[^ ^27^ ^]^ an Ago2 inhibitor, was increased (Figure 6D). Ago2 is a key component of the RNA‐induced silencing complex responsible for mRNA degradation and gene expression regulation in the cytoplasm.^[^ ^28^ ^]^ After treating Neuro2a cells transfected with ADV‐shCtrl and ADV‐shFam172a with actinomycin D (a transcriptional inhibitor)^[^ ^29^ ^]^ at different time points, we found that knockdown of Fam172a promoted the degradation of Oxt gene mRNA, while BCI‐137 treatment effectively prolonged its mRNA lifetime of Oxt gene (Figure 6E). In summary, reduced Fam172a expression in neurons elevated cytoplasmic Ago2 levels, accelerating mRNA degradation and decreasing Oxt release.

### 2.8 Ago2 Specific Knockdown and Oxt Administration Effectively Ameliorate Anxiety‐Like Behaviors in OFKO Mice

**Figure 7:**
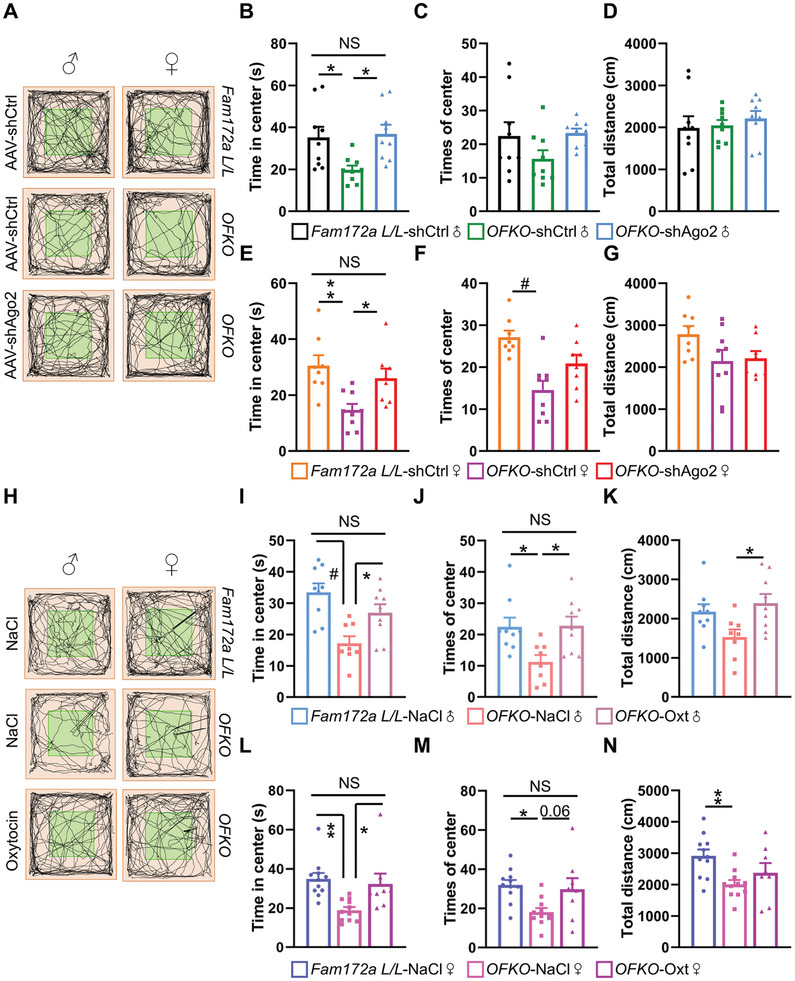
The specific knockdown of Ago2 or administration of Oxt alleviates anxiety‐like behaviors in *OFKO* mice. A) The PVN of *OFKO* mice was injected with the knockdown virus AAV‐FLEX‐shAgo2‐EGFP (*OFKO*‐shAgo2), while a control virus (AAV‐FLEX‐shCtrl‐EGFP) was administered to both *Fam172a L/L* mice (*Fam172a L/L*‐shCtrl) and another group of *OFKO* mice (*OFKO*‐shCtrl) in the same region. The activity trajectory line plot was used to measure anxiety‐like behavior in *Fam172a L/L*‐shCtrl mice, *OFKO*‐shCtrl mice, and *OFKO*‐shAgo2 mice. B–D) Time in center (B), times of center (C), and total distance (D) were assessed in the OFT for *Fam172a L/L*‐shCtrl male mice, *OFKO*‐shCtrl male mice, and *OFKO*‐shAgo2 male mice. *n* = 9 (*Fam172a L/L*‐shCtrl, male), 9 (*OFKO*‐shCtrl, male), or 9 (*OFKO*‐shAgo2, male) mice per group. E–G) Time in center (E), times of center (F), and total distance (G) were assessed in the OFT for *Fam172a L/L*‐shCtrl female mice, *OFKO*‐shCtrl female mice, and *OFKO*‐shAgo2 female mice. *n* = 8 (*Fam172a L/L*‐shCtrl, female), 9 (*OFKO*‐shCtrl, female), or 8 (*OFKO*‐shAgo2, female) mice per group. H) Anxiety‐like behavior was measured in *Fam172a L/L* and *OFKO* mice treated with NaCl or Oxt under chow diet conditions. The activity trajectory line plot. I–K) Time in the center (I), times of center (J), and total distance (K) were assessed in the OFT for NaCl‐treated *Fam172a L/L* male mice, NaCl‐treated *OFKO* male mice, and Oxt‐treated *OFKO* male mice. *n* = 8 (*Fam172a L/L*‐NaCl, male), 8 (*OFKO*‐NaCl, male), or 9 (*OFKO*‐Oxt, male) mice per group. L–N) Time in the center (L), times of center (M), and total distance (N) were assessed in the OFT for NaCl‐treated *Fam172a L/L* female mice, NaCl‐treated *OFKO* female mice, and Oxt‐treated *OFKO* female mice. *n* = 9 (*Fam172a L/L*‐NaCl, female), 10 (*OFKO*‐NaCl, female), or 8 (*OFKO*‐Oxt, female) mice per group. The data in (B), (E), (F), (I) to (K), and (L) to (N) are presented as mean ± SEM. ^*^ *p* < 0.05, ^**^ *p* < 0.01, #*p* < 0.001, *p*‐values are calculated using one‐way ANOVA with Tukey correction (B‐G, I‐N).

To validate our results, we created a Cre‐inducible AAV (AAV‐FLEX‐shAgo2‐EGFP) and injected it into the PVN of *OFKO* mice (*OFKO*‐shAgo2). As controls, a virus (AAV‐FLEX‐shCtrl‐EGFP) was administered to *Fam172a L/L* (*Fam172a L/L*‐shCtrl) and *OFKO* (*OFKO*‐shCtrl) mice in the same region. Immunofluorescence assays confirmed the specific expression of the virus and precision of the injection sites (Figure S9A, Supporting Information). Western blot analysis demonstrated reduced Ago2 expression in the hypothalamus of *OFKO*‐shAgo2 mice, validating the effectiveness of the viral construct (Figure S9B,C, Supporting Information). At week 16, *OFKO*‐shAgo2 males exhibited improvement, as anxiety tests revealed that they spent more time in the center zone of the OFT compared to *OFKO*‐shCtrl males (**Figure** **7**A,B), and no significant differences were observed among the three groups in terms of center entries (Figure 7C) or the total distance traveled (Figure 7D). Consistent with the findings in male mice, the anxiety levels in *OFKO*‐shAgo2 mice were demonstrated an improvement (Figure 7A). The *OFKO*‐shAgo2 female spent more time in the center zone (Figure 7E), yet there were no statistically significant differences in terms of frequency of entries (Figure 7F) and the total distance traveled (Figure 7G). In summary, targeted downregulation of Ago2 in *OFKO* mice is found to improve anxiety behaviors irrespective of gender. The results show reducing Ago2 expression in PVN Oxt neurons alleviates anxiety induced by Fam172a deficiency, thereby implicating Ago2 as a modulator of this response.

Additionally, we performed behavioral assessments on *Fam172a L/L* or *OFKO* mice 30 min following intranasal administration of either NaCl or Oxt.^[^ ^30^ ^]^ In the OFT (Figure 7H), Oxt administration alleviated anxiety‐like behavior in *OFKO* mice, regardless of sex. Specifically, compared to NaCl‐administered *OFKO* mice, those administered with Oxt spent more time and made more entries in a central zone, traveling farther distances (Figure 7I–N).

## 3 Discussion

Obesity is a prevalent social issue, with steadily increasing prevalence rates. It is a multifactorial disease primarily caused by dietary factors, and HFD is a major contributor to obesity. Diet‐induced obesity can lead to mental health problems, particularly anxiety disorders.^[^ ^3^, ^31^ ^]^ Studies have reported that DIO disrupts the balance of the nervous system, contributing to the development of anxiety disorders.^[^ ^22^ ^]^ Interestingly, studies have shown that neurological impairments and metabolic phenotypes induced by chronic high‐fat feeding in mice are associated with anxiety‐like behavior, regardless of changes in body weight,^[^ ^32^ ^]^ which aligns with our findings.

The Oxt neuronal system in the hypothalamic PVN has been shown to be closely linked to anxiety disorders.^[^ ^13^ ^]^ Studies have highlighted the role of hypothalamic Oxt neurons in modulating anxiety states.^[^ ^8^, ^24^ ^]^ Oxt is a peptide hormone secreted by the hypothalamic Oxt neurons; it has been found to exhibit stress and anxiety resilience^[^ ^33^ ^]^ while ameliorating anxiety symptoms in DIO.^[^ ^7^ ^]^ Interestingly, reduced Oxt serum concentrations have been observed in obesity induced by a HFD, and Oxt treatment has been shown to improve this impaired state.^[^ ^34^ ^]^ Oxt has also been utilized as an effective treatment for individuals with anxiety disorders.^[^ ^11a^ ^]^ In this study, we discovered a decrease in blood Oxt concentration in a mouse model of anxiety induced by CRS.

To gain deeper insights into how the Oxt neuronal system regulates anxiety‐like behavior, based on the 28 differential genes identified in a previous study on DNA methylation in whole blood cell sequencing of Han Chinese patients with anxiety disorders, we identified these genes that exhibited differential methylation.^[^ ^14^ ^]^ We conducted these gene screenings of the hypothalamus in mouse models fed with either CD or HFD for eight weeks (HFD 8wks). In these genes, Fam172a was noted for its reduction in expression within the group of HFD 8wks mice. The expression of Fam172a in the hypothalamus was found to be negatively correlated with anxiety levels, which was confirmed through immunofluorescence staining. Fam172a is a highly conserved protein.^[^ ^26^ ^]^ We conducted immunofluorescence staining of brain slices with ARS mice and found that both Fam172a expression and Oxt neuronal activity decreased in the PVN under the HFD condition. In our study, through specific knockout mouse models of Fam172a, we determined that the absence of Fam172a in Oxt neurons leads to increased anxiety levels and decreased blood Oxt concentration. However, this can be rescued by nasal Oxt treatment. Conversely, overexpression of Fam172a in mice effectively alleviated anxiety symptoms induced by a HFD regimen, leading to an increase in blood Oxt concentration. Notably, we did not observe significant changes in body weight and glucose tolerance in mice. Collectively, our findings highlight the importance of Fam172a as a key protein involved in Oxt synthesis and release.

Among the Argonaute family proteins, Ago2 is the only catalytically active protein closely associated with mRNA degradation.^[^ ^35^ ^]^ Ago2 plays a crucial role as an essential component of the mRNA‐induced silencing complex and is primarily localized in cytoplasmic bodies.^[^ ^28^ ^]^ Research has revealed that Fam172a acts as a central player in Ago2 functioning.^[^ ^26^ ^]^ According to the latest findings, Fam172a directly participates in the nuclear movement of Ago2 through a nuclear localization signal.^[^ ^20b^ ^]^ Therefore, we investigated whether Fam172a promotes mRNA decay by facilitating Ago2 entry into the nucleus. Our results indicated that overexpression of Fam172a facilitated the entry of Ago2 into the cytoplasm, leading to accelerated degradation of Oxt gene mRNA. These findings present new prospects for future therapeutic targets, although further investigation is required to elucidate the underlying molecular mechanisms.

In conclusion, our study suggests that Fam172a in Oxt neurons of the hypothalamic PVN impacts Ago2 expression in both the nucleus and cytoplasm, thereby altering the stability of Oxt mRNA. Thereby influencing Oxt release and modulating anxiety levels in obese mice.

## 4 Experimental Section

### Animals

Male and female *C57BL/6* mice were purchased from Gempharmatech Co., Ltd (Nanjing, China). *Fam172a^loxP/loxP^* mice were provided by Dr. Lianxi Li (Shanghai Sixth People's Hospital, affiliated with Shanghai Jiao Tong University School of Medicine). The *Oxt^Cre^ Rosa26‐tdTomato* mice were generated by crossing the *Oxt^Cre^* with *Rosa26‐tdTomato* mice, and the *Oxt^Cre^ Fam172a^loxP/loxP^* mice by crossing the *Oxt^Cre^* with the *Fam172a^loxP/loxP^* mice. Regular chow (9.4% kcal from fat) and HFD (60% kcal from fat) were purchased from Xietong Bioscience (Beijing, China) and Research Diets (New Brunswick, NJ, USA), respectively. All mice were housed in a constant temperature (22–24 °C) chamber in a 12‐h light/dark circadian rhythm, and provided with adequate water and food. The mice were randomly assigned to different experimental groups and were tested by an experimenter who was blind to their grouping. All experimental procedures were approved by the Institutional Animal Care and Use Committee of Shanghai Sixth People's Hospital Affiliated with Shanghai Jiao Tong University School of Medicine (DWSY2022‐0049).

### Cell Culture

Neuro2a cells were cultured in Dulbecco's modified Eagle's medium (DMEM) with 10% fetal bovine serum (FBS) containing antibiotics.^[^ ^36^ ^]^ The cells were transfected with ADV‐shRNA‐EGFP and ADV‐shFam172a‐EGFP, each sample having a titer of 10^8^ (v.g. mL^−1^) and incubated for 48 h at 37 °C. Subsequently, the cells were harvested. Total protein was extracted and used for protein blot analysis. ADV‐shRNA‐EGFP and ADV‐shFam172a‐EGFP were manufactured by OBiO Technology Shanghai Corp. Ltd. (Shanghai, China)

To test the effect of Fam172a on mRNA stability, 4 (µg mL^−1^) actinomycin D (MCE, HY‐17559) was added to Neuro2a cells 24 h after transfection with ADV‐shRNA‐EGFP and ADV‐shFam172a‐EGFP and RNA was prepared by collecting samples at 0, 15, 45, and 90 min time points thereafter. The relative amount of Oxt and β‐actin mRNA was determined by RT‐PCR and normalized to Oxt mRNA. The 0 min Oxt mRNA relative amount was 100%, and the amount at the later time points was reported as a percentage of the 0 min time point.^[^ ^17^, ^37^ ^]^

### Immunofluorescence

Briefly, the mice were anesthetized using avertin (300mg kg^−1^), the heart was exposed, and normal saline was perfused for 5 min, followed by 4% paraformaldehyde (PFA) solution for 15 min. After removing the mouse brains, it was fixed with 4% PFA and dehydrated with a gradient of 20% and 30% sucrose, respectively. Brain tissues were divided into 25 µm thick sections using a freezing microtome. The tissue sections were first soaked in a PBS solution for 5 min, and then incubated in a PBS solution containing 5% serum and 0.3% Triton X‐100 for 30 min, followed by rabbit anti‐Fam172a (1:1000, Abcam, ab121364), rabbit anti‐c‐Fos (1:1000, sysy, 226008), and guinea pig anti‐c‐Fos (1:2000, sysy, 226017) overnight at 4 °C. They were incubated with the Alexa Fluor 555 goat anti‐pig, Alexa Fluor 633 or 488 goat anti‐rabbit, and Alexa Fluor 555 donkey anti‐rabbit secondary antibodies (1:800, Thermo Fisher, Waltham, MA) at room temperature for 1 h. DAPI dye was used to label cell nuclei and images were acquired using an LSM980 confocal microscope (Carl Zeiss, Jena, Germany). For each mouse, cells on the nuclear side of the PVN were manually counted in representative images.

The method for cellular immunofluorescence has been described elsewhere.^[^ ^38^ ^]^ Briefly, Neuro2a cells were transfected with ADV‐shRNA‐EGFP and ADV‐shFam172a‐EGFP for 48 h after treatment. Cells on glass coverslips were fixed in 4% PFA for 10 min at room temperature. Subsequently, cells were blocked using a PBS solution containing 5% serum and 0.3% Triton X‐100 for 30 min prior to immunofluorescence staining for Ago2 (1:1000, Abcam, ab186733), and images were acquired using an LSM980 confocal microscope (Carl Zeiss, Jena, Germany) and polarized structured illumination microscopy. Images were processed via ImageJ (NIH, Bethesda, MD).

### Stereotaxic Surgery

The mice were anesthetized using avertin (300mg kg^−1^) and placed on a stereotaxic instrument. The adeno‐associated virus was injected into bilateral PVN (coordinates, RC: −0.085 cm, ML: ±0.025 cm, DV: −0.48 cm), and the (400 nL) virus was injected at a rate of 0.1 (µL min^−1^) using a microsyringe pump. All AAV viruses were produced by OBiO Technology Shanghai Corp. Ltd. (Shanghai, China) or Shanghai Genechem Co. Ltd. (Shanghai, China) and had titers of >10^12^ (v.g. mL^−1^). Each injection utilized (0.4 µL) of AAV and lasted for 5 min. Behavioral experiments were performed 1 week after the surgery. For pharmacogenetic manipulations, CNO (MCE, HY‐17366A) was dissolved in sterile saline and injected intraperitoneally at a dosage of 1 (mg kg^−1^) body weight.^[^ ^39^ ^]^ The intraperitoneal injection (IP) was performed 2 h prior to the desired experimental procedure. The mice were allowed a recovery period of one week prior to the initiation of any experimental procedures.

### Quantitative RT‐PCR

Total RNA was extracted using TRIzol reagent (Thermo Fisher) in hypothalamic tissue. An RT reagent kit (Takara) was used to reverse transcribe the mRNA to obtain cDNA. Using the QuantStudio 7 Flex Real‐Time Fluorescent Quantitative PCR System (Thermo Fisher), RT‐PCR was performed using a (10 µL) mix containing cDNA, primers, and SYBR Green Premix (Thermo Fisher). Using the 2^−ΔCt^ method, was used to calculate the final results of relative mRNA levels, where ΔCt was the difference between the Ct value of the target gene and β‐actin control. Table S1 (Supporting Information) presents the details of the primer sequences.

### Western Blot

The total proteins were extracted from the Neuro2a cells transfected with ADV‐shRNA‐EGFP and ADV‐shFam172a‐EGFP, separated by SDS‐PAGE, and transferred to the PVDF membranes. After sealing the membrane with 5% skim milk, they were incubated overnight with rabbit anti‐Fam172a (1:500), rabbit anti‐Ago2 (1:2000), or rabbit anti‐β‐actin (1:2000) at 4 °C. After incubation with horseradish peroxidase‐conjugated secondary antibody (1:10 000), the membrane was exposed to the Supersignal West Femto Maximum Sensitivity Substrate (Thermo Fisher) and chemiluminescence was recorded in the GeneGnome system (Syngene, Cambridge, UK).

### Restraint Stress

The homemade binding mold was made of a (50 mL) syringe as the main body, and ten small holes of uniform size were randomly drilled around the tube wall to ensure that the mouse could breathe. The control group mice were placed in the same environment as the experimental group during the restraint period, with food and water fasting. Mice in the experimental group were placed in restraint devices that ensured smooth breathing and immobility. This restraint was consistently administered for three days (ARS) or seven days (CRS). Between 9:00 AM and 12:00 PM, the mice were confined to the same tube daily and restrained for 2 h to observe their respiration and physical state during restraint. At the end of the test, the restraint devices were cleaned with clean water, followed by the use of 75% alcohol to remove odors.^[^ ^23a^ ^]^

### Anxiety‐Like Behavioral Analysis

Before the behavioral test, the experimental mice were placed in the behavioral room for 30 min to exclude the influence of environmental factors such as light and sound.^[^ ^40^ ^]^ The models were cleaned before and after each experiment.

### Open Field Test

At the beginning of the experiment, the mice were placed in the center of an open opaque cube box (40 cm × 40 cm × 40 cm) and the camera located at the top of the maze was immediately activated to record the distance the mice moved and the time and number of times they entered the center region, for 5 min.^[^ ^41^ ^]^ The cabinet was divided into a central area (10 cm × 10 cm) and a surrounding area, which were analyzed and processed by Tracking master 3.0 (Fanbi Intelligent Technology Co, China).

### Elevated Plus‐Maze Test

The maze consisted of two open arms (30 cm L × 5 cm W) and closed arms (30 cm L × 5 cm W × 18 cm H) and was 60 cm above the ground. Mice were placed in the center region of the model and their activity was observed by a camera located directly above, for 5 min.^[^ ^41^ ^]^

### Elevated Zero Maze Test

The labyrinth was a circular platform located (65 cm) above the ground (runway 6 cm W, outer diameter 50 cm, and inner diameter 38 cm). The maze was symmetrically divided into two open and closed zones (14 cm medial height and 17 cm lateral height). The mice were placed in the middle of an arbitrary open area with their heads facing the center of the circle and their behavioral changes were recorded with a camera for 5 min.^[^ ^42^ ^]^

### Oxt Assays

At the conclusion of the trial, blood was collected from the mice and centrifuged to obtain plasma. Additionally, culture media were harvested from Neuro2a cells 24 h post‐transfection with ADV‐shRNA‐EGFP and ADV‐shFam172a‐EGFP. Plasma Oxt was measured using the Oxytocin Elisa kit (Enzo, Farmingdale, NY, USA).

### Oxt Administration

Oxt (Sangon, A605015) was dissolved in a solution of PBS and administrated intranasally in a dose of 200 (µg kg^−1^), as previously reported.^[^ ^43^ ^]^ Briefly, the mice were given Oxt and PBS intranasally, as a control 30 min prior to behavioral experiments. A P10 pipette was used to inject 100 (µg kg^−1^) Oxt or an equal volume of control solution into each nostril.

### Statistical Analysis

Data were presented as mean ± SEM. The Figure legend indicates the exact number of mice and cultures used in the experiment. The data were analyzed using the two‐tailed Student's *t*‐test or one‐way and two‐way ANOVA followed by Bonferroni's or Tukey's post hoc test. When P<0.05, the data were considered statistically significant. Table S2 (Supporting Information), Information with the inclusion of P values and F statistics. Statistical analysis was performed using Prism 9 (GraphPad Software, San Jose, CA).

## Conflict of Interest

The authors declare no conflict of interest.

## Author Contributions

B.W., L.Z., and X.W. contributed equally to this work. C.H., Z.C., and Y.Z. designed the study. B.W. conducted the experiments, analyzed the data, and wrote the manuscript. Z.C. and C.H. helped analyze the data and revised the manuscript. L.Z. assisted with the experiments. R.Z., Y.Z., and L.L. provided helpful suggestions regarding the experiments. C.Z. and C.H. directed the research.

## Supporting information



Supporting Information

## Data Availability

The data that support the findings of this study are available from the corresponding author upon reasonable request.
