# Supplementary material for: Fam172a Mediates the Stimulation of Hypothalamic Oxytocin Neurons to Suppress Obesity‐Induced Anxiety
Source: Adv Sci (Weinh). 2025 Feb 17;12(14):2414723. doi: 10.1002/advs.202414723 (PMC11984834; doi:10.1002/advs.202414723)
Supplement: Supplementary file 1 — Supporting Information [file ADVS-12-2414723-s001.pdf]

ADVANCED SCIENCE
Open Access

Supporting Information

for *Adv. Sci.*, DOI 10.1002/advs.202414723

Fam172a Mediates the Stimulation of Hypothalamic Oxytocin Neurons to Suppress Obesity-Induced Anxiety

Baocheng Wan, Lina Zhang, Xinyu Wang, Rong Zhang, Lianxi Li, Yi Zhang*, Zhuo Chen* and Cheng Hu**

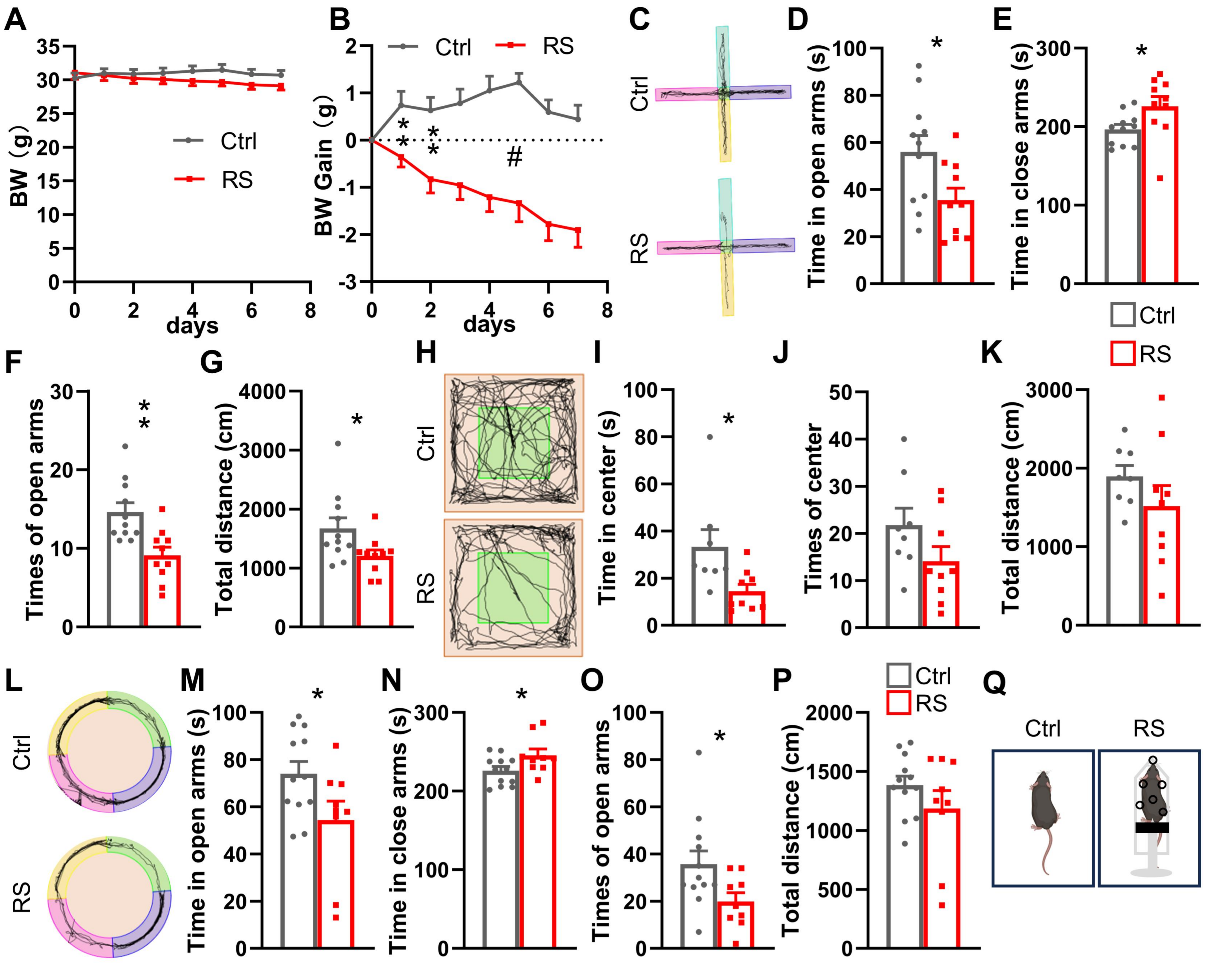

Figure S1. RS leads to anxiety-like behavior and inhibition in Oxt neuronal activity in the PVN.

(A and B) Adult male *C57BL/6* mice were fed a chow diet, and body weight (A) and weight gain (B) was recorded for 7 consecutive days in the RS experiment. n = 9 (Ctrl) or 10 (RS) mice per group.

(C–G) Activity trajectory line plot (C) of Ctrl mice and RS mice, time in open arms (D), time in close arms (E), times of open arms (F), and total distance (G) in the EPM. n = 11 (Ctrl) or 10 (RS) mice per group.

(H–K) Activity trajectory line plot (H), time in center (I), times in center (J), and total distance (K) during the OFT. n = 8 (Ctrl) or 9 (RS) mice per group.

(L–P) Activity trajectory line plot (L), time in open arms (M), time in close arms (N), times of open arms (O), and total distance (P) during the EZM. n = 12 (Ctrl) or 9 (RS) mice per

group.

(Q) Schematic diagram of restraint stress mouse model. Created in BioRender. Chen, Z. (2024) https://BioRender.com/m15b213.

The data in (B), (D) to (G), (I), and (M) to (O) are presented as mean ± SEM.

*P < 0.05; **P < 0.01; #P < 0.001, P-values are calculated using two-way ANOVA with Bonferroni correction (A, B), two-tailed Student's t-test (D-G, I-K, M-P).

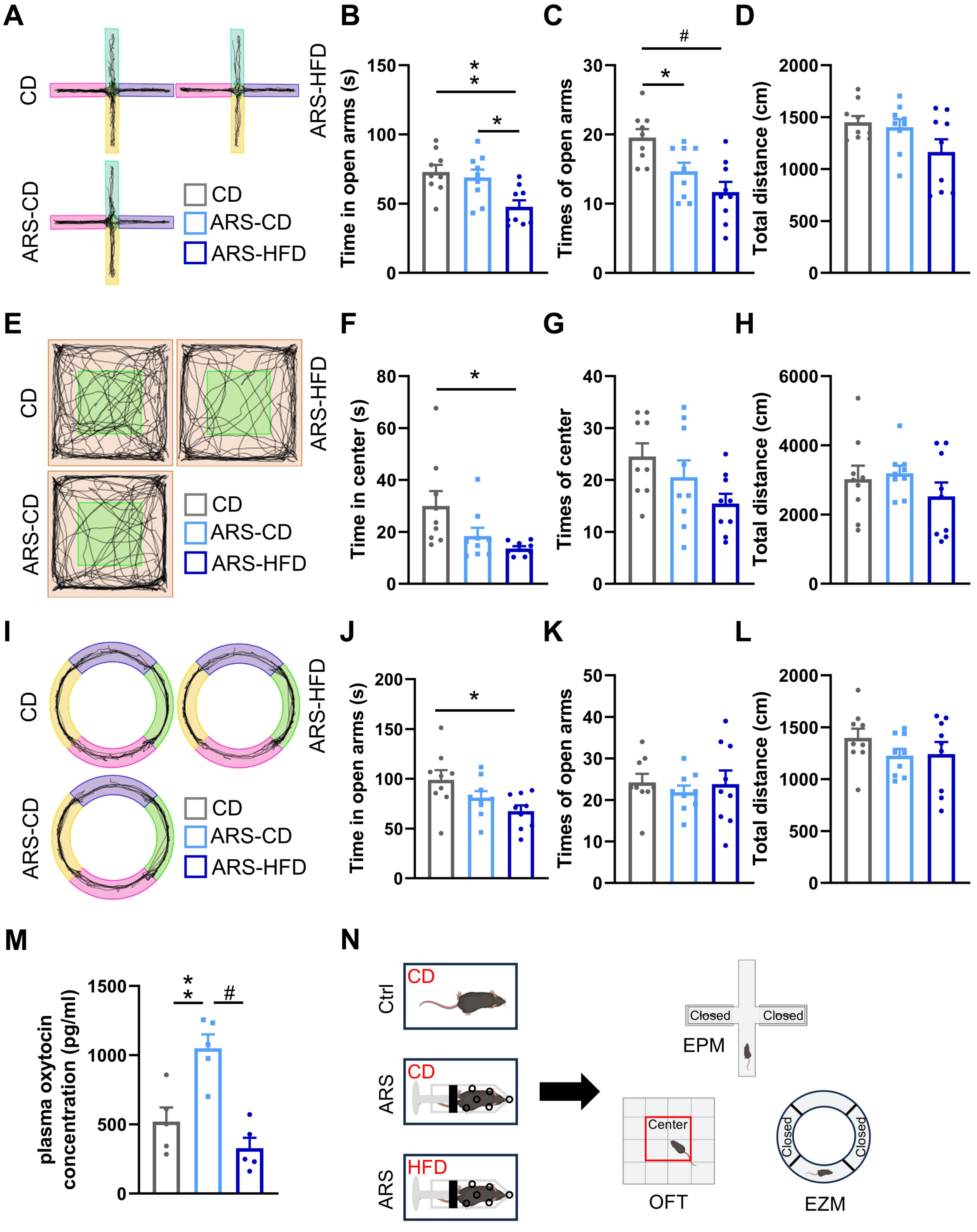

Figure S2. During periods of stress, HFD promotes anxiety development and decreases Oxt concentration in male mice.

(A–D) To assess the anxiety-like behavior of male mice in the CD, ARS-CD, and ARS-HFD groups, in the elevated plus maze (EPM), we evaluated the activity trajectories of mice (A),

measuring the time in open arms (B), times of open arms (C), and total distance (D). n= 9 (CD), 9 (ARS-CD), or 9 (ARS-HFD) mice per group.

(E–H) We utilized the open field test (OFT) to evaluate their activity tracks (E), time in center (F), times of center (G), and total distance (H). n= 9 (CD), 9 (ARS-CD), or 9 (ARS-HFD) mice per group.

(I–L) The mice activity trajectory line plot (I), time in open arms (J), times of open arms (K), and total distance (L) were assessed during the EZM. n = 9 (CD), n = 9 (ARS-CD), or 9 (ARS-HFD) mice per group.

(M) Plasma was extracted from CD, ARS-CD, or ARS-HFD mice, and plasma Oxt levels were determined. n = 5 for each group.

(N) Schematic representation of the experimental design used for establishing the CD, ARS-CD, and ARS-HFD conditions. Created in BioRender. Chen, Z. (2024) https://BioRender.com/m15b213.

The data in (B), (C), (F), (J), and (M) are presented as mean ± SEM.

*P < 0.05, **P < 0.01, #P < 0.001, P-values are calculated using one-way ANOVA with Tukey correction (B-D, F-H, J-M).

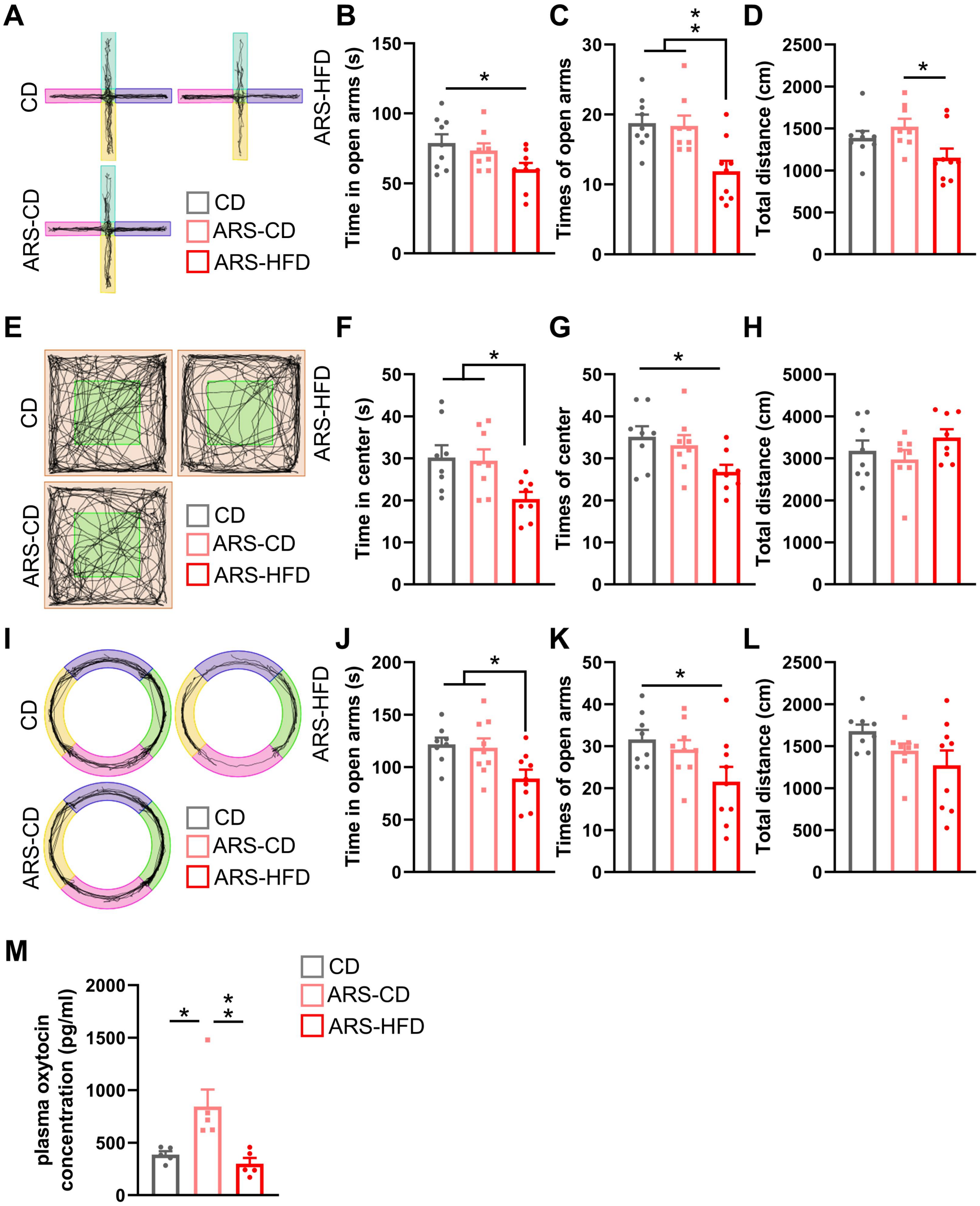

Figure S3. During periods of stress, HFD promotes anxiety development and decreases Oxt concentration in female mice.

(A–D) To assess the anxiety-like behavior of female mice in the CD, ARS-CD, and ARS-HFD groups, we utilized the EPM. We evaluated the activity trajectories of mice (A),

measuring the time in open arms (B), times of open arms (C), and total distance (D). n= 9 (CD), 8 (ARS-CD), or 9 (ARS-HFD) mice per group.

(E–H) In the OFT, we evaluated the mice activity tracks (E), time in center (F), times of center (G), and total distance (H). n= 8 (CD), 8 (ARS-CD), or 8 (ARS-HFD) mice per group.

(I–L) The mice activity trajectory line plot (I), time in open arms (J), times of open arms (K), and total distance (L) were assessed during the EZM. n = 8 (CD), 9 (ARS-CD), or 9 (ARS-HFD) mice per group.

(M) Plasma was extracted from CD, ARS-CD, or ARS-HFD mice, and plasma Oxt levels were determined. n = 5 for each group.

The data in (B) to (D), (F) to (G), (J) to (K), and (M) are presented as means ± SEM. $*P < 0.05$, $**P < 0.01$, P-values are calculated using one-way ANOVA with Tukey correction (B-D, F-H, J-M).

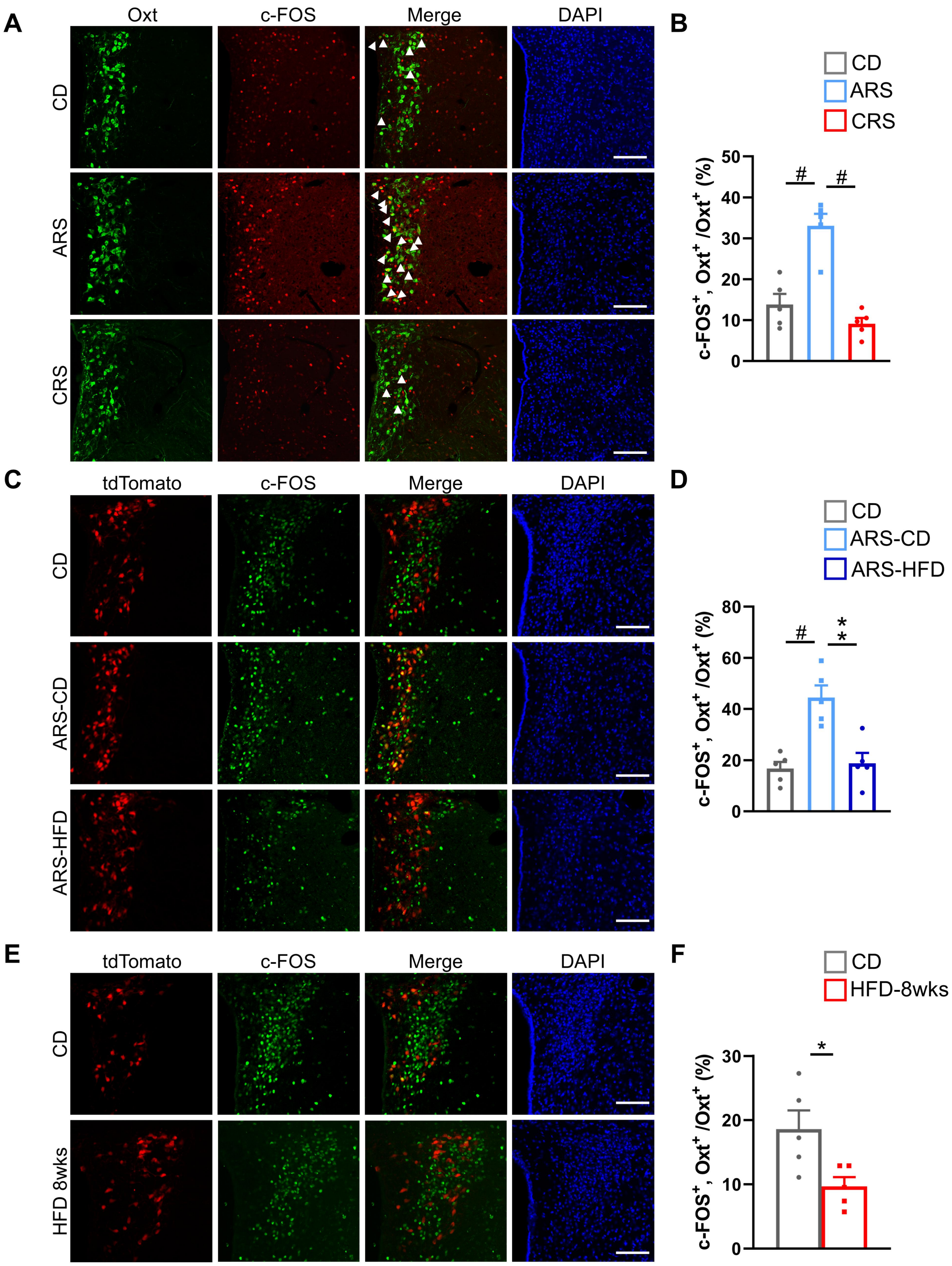

Figure S4. HFD reduces Oxt neuronal activity in the PVN of mice.

(A) Double immunofluorescence staining of Oxt (green) and c-Fos (red) was performed on brain sections of CD, ARS, or CRS mice. Cell nuclei were counter-stained with DAPI (blue). Arrows indicate Oxt and c-Fos co-expressing cells. Scale bars, 100 μm.

(B) Percentage of Oxt cells expressing c-Fos in the PVN of CD, ARS, or CRS mice. n = 5 (CD), 5 (ARS), or 5 (CRS) mice per group.

(C) Brain sections of the CD, ARS-CD, or ARS-HFD *Oxt*^{Cre} *Rosa* mice were immunostained for c-Fos (green). Cell nuclei were counter-stained with DAPI (bule). Scale bars, 100 μm.

(D) Percentage of Oxt cells expressing c-Fos in the PVN of CD, ARS-CD, or ARS-HFD mice. n = 5 (CD), 5 (ARS-CD), or 5 (ARS-HFD) mice per group.

(E) Immunofluorescence staining of c-Fos (green), a neuronal activity marker, was performed on brain sections of CD *Oxt*^{Cre} *Rosa* mice or HFD 8wks *Oxt*^{Cre} *Rosa* mice. Cell nuclei were counter-stained with DAPI (blue). Scale bars, 100 μm.

(F) Percentage of Oxt cells expressing c-Fos in the PVN of CD or HFD 8wks mice. n = 5 (CD) or 5 (HFD 8wks) mice per group.

The data in (B), (D), and (F) are presented as means ± SEM.

*P < 0.05, **P < 0.01, #P < 0.001, P-values are calculated using one-way ANOVA with Tukey correction (B, D), two-tailed Student's t-test (F).

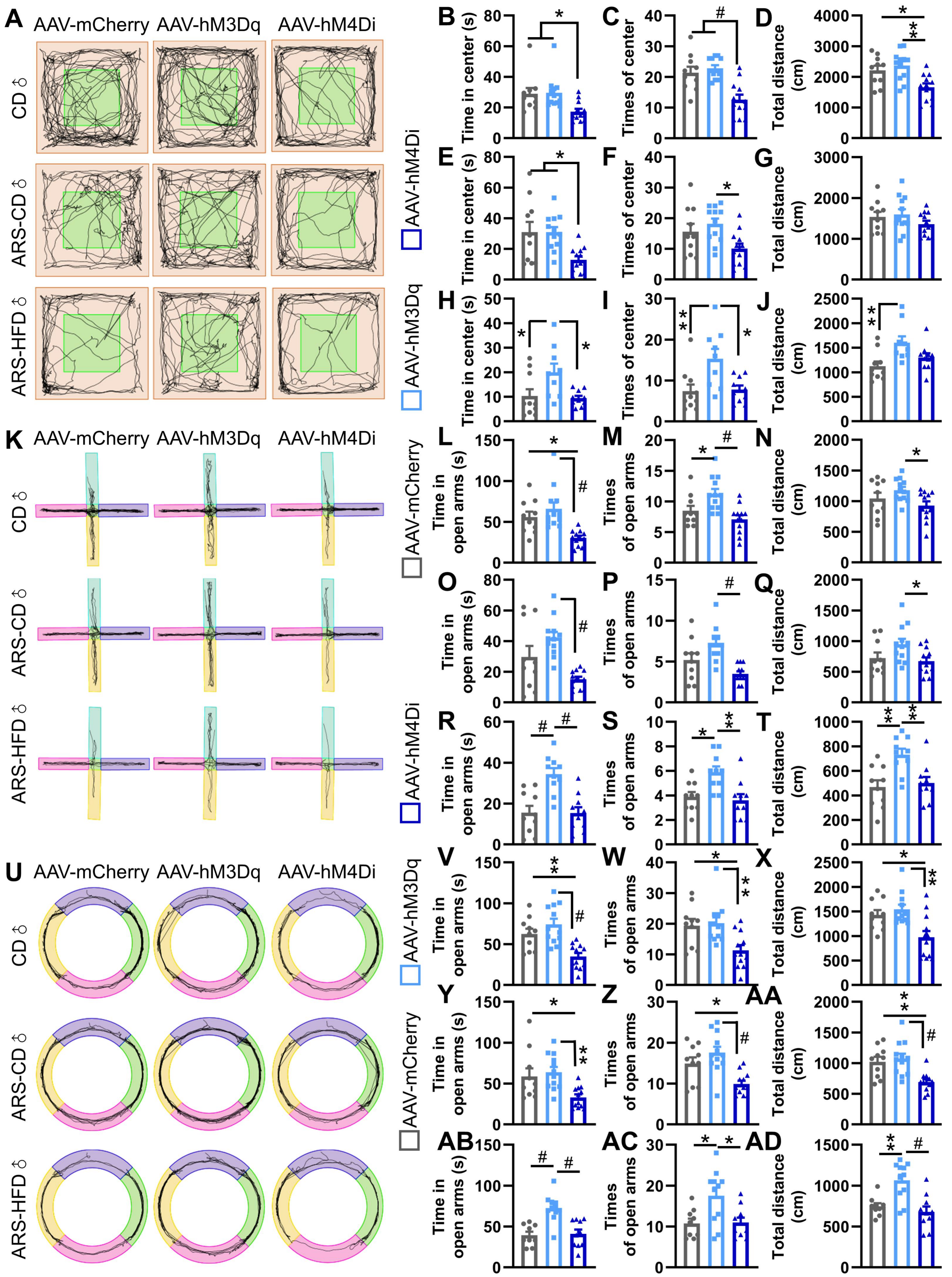

Figure S5. The neuronal activity of Oxt in the PVN of male mice regulates the progression of anxiety-like behaviors.

(A–J) 8-week-old male *Oxt*^{Cre} male mice were injected with Cre inducible AAV-mCherry,

AAV-hM3Dq, and AAV-hM4Di viruses into the PVN and fed a CD for 8 weeks. Before conducting the anxiety assessments, male mice were subjected to CD, ARS-CD, and ARS-HFD treatments. To assess anxiety-like behavior in male mice in the CD, ARS-CD, and ARS-HFD groups, we utilized the OFT to evaluate their activity tracks (A); time in center (B), (E), (H); times of center (C), (F), (I); and total distance (D), (G), (J).

n = 10 (CD-AAV-mCherry), 12 (CD-AAV-hM3Dq), 12 (CD-AAV-hM4Di), 10 (ARS-CD-AAV-mCherry), 12 (ARS-CD-AAV-hM3Dq), 12 (ARS-CD-AAV-hM4Di), 10 (ARS-HFD-AAV-mCherry), 9 (ARS-HFD-AAV-hM3Dq), or 10 (ARS-HFD-AAV-hM4Di) mice per group.

(K–T) In the EPM, we evaluated the activity trajectories of mice (K), measuring the time in open arms (L), (O), (R); the times of open arms (M), (P), (S); and total distance (N), (Q), (T).

n = 10 (CD-AAV-mCherry), 12 (CD-AAV-hM3Dq), 12 (CD-AAV-hM4Di), 10 (ARS-CD-AAV-mCherry), 12 (ARS-CD-AAV-hM3Dq), 12 (ARS-CD-AAV-hM4Di), 10 (ARS-HFD-AAV-mCherry), 10 (ARS-HFD-AAV-hM3Dq), or 10 (ARS-HFD-AAV-hM4Di) mice per group.

(U–AD) The mice activity trajectory line plot (U); time in open arms (V), (Y), (AB); times of open arms (W), (Z), (AC); and total distance (X), (AA), (AD) were assessed during the EZM.

n = 10 (CD-AAV-mCherry), 12 (CD-AAV-hM3Dq), 12 (CD-AAV-hM4Di), 10 (ARS-CD-AAV-mCherry), 12 (ARS-CD-AAV-hM3Dq), 12 (ARS-CD-AAV-hM4Di), 9 (ARS-HFD-AAV-mCherry), 11 (ARS-HFD-AAV-hM3Dq), or 10 (ARS-HFD-AAV-hM4Di) mice per group.

The data in (B) to (F), (H) to (J), (L) to (T), and (V) to (AD) are presented as mean ± SEM.

*$P < 0.05$, **$P < 0.01$, #$P < 0.001$, P-values are calculated using one-way ANOVA with Tukey correction (B-J, L-T, V-AD).

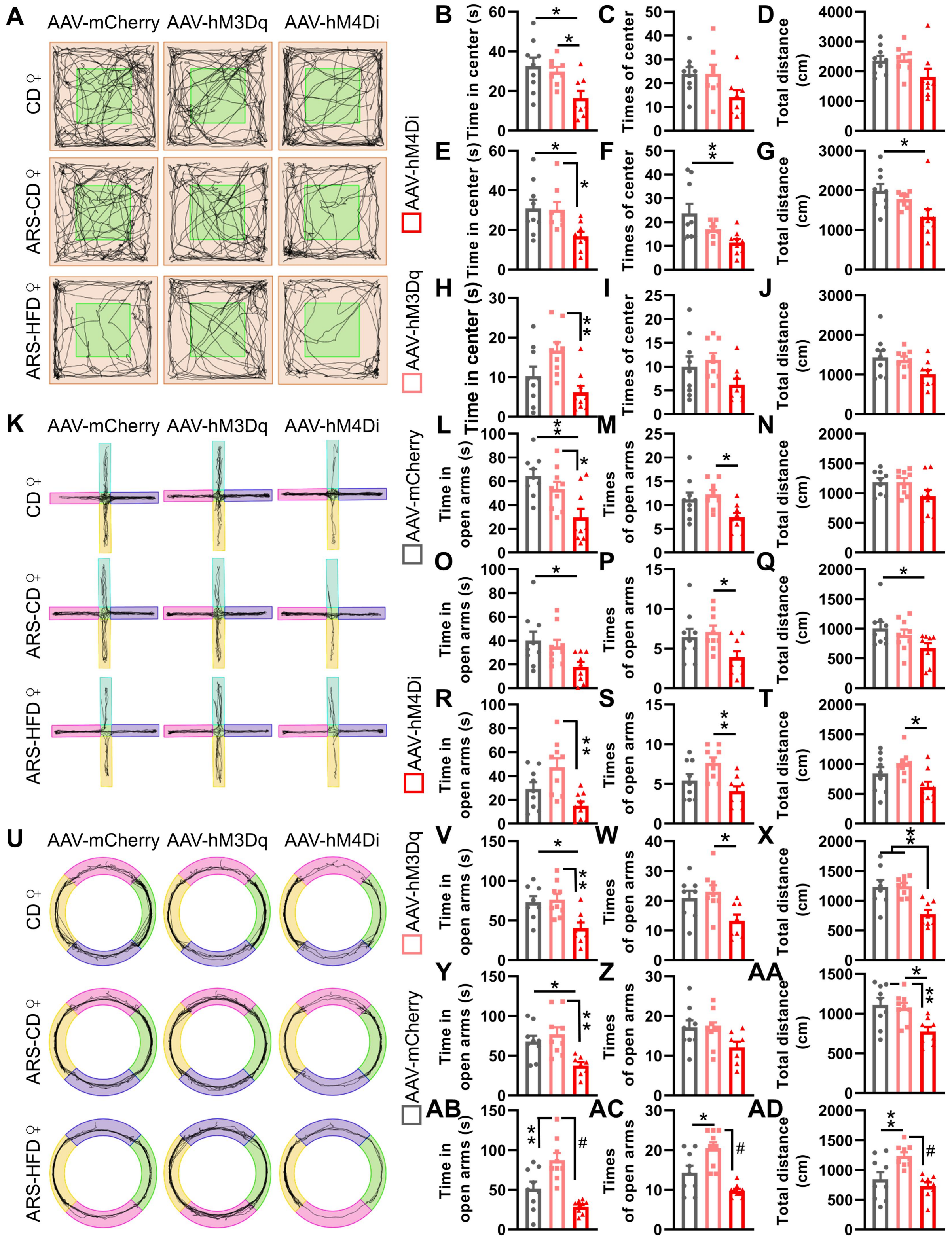

Figure S6. Oxt neuronal activity in the PVN of female mice regulates the development of anxiety-like behaviors.

(A–D) 8-week-old *Oxt*^{Cre} female mice were injected with Cre inducible AAV-mCherry, AAV-hM3Dq, and AAV-hM4Di viruses into the PVN and fed a CD for 8 weeks. Before conducting

the anxiety assessments, female mice were subjected to CD, ARS-CD, and ARS-HFD treatments. To assess anxiety-like behavior in female mice in the CD, ARS-CD, and ARS-HFD groups, we utilized the OFT to evaluate their activity tracks (A), time in center (B), (E), (H); times of center (C), (F), (I); and total distance (D), (G), (J).

n = 9 (CD-AAV-mCherry), 8 (CD-AAV-hM3Dq), 8 (CD-AAV-hM4Di), 9 (ARS-CD-AAV-mCherry), 8 (ARS-CD-AAV-hM3Dq), 9 (ARS-CD-AAV-hM4Di), 9 (ARS-HFD-AAV-mCherry), 9 (ARS-HFD-AAV-hM3Dq), or 9 (ARS-HFD-AAV-hM4Di) mice per group.

(K–T) In the EPM, we evaluated the activity trajectories of mice (K); measuring the time in open arms (L), (O), (R); the times of open arms (M), (P), (S); and total distance (N), (Q), (T).

n = 9 (CD-AAV-mCherry), 9 (CD-AAV-hM3Dq), 9 (CD-AAV-hM4Di), 9 (ARS-CD-AAV-mCherry), 9 (ARS-CD-AAV-hM3Dq), 9 (ARS-CD-AAV-hM4Di), 9 (ARS-HFD-AAV-mCherry), 9 (ARS-HFD-AAV-hM3Dq), or 9 (ARS-HFD-AAV-hM4Di) mice per group.

(U–AD) The mice activity trajectory line plot (U); time in open arms (V), (Y), (AB); times of open arms (W), (Z), (AC); and total distance (X), (AA), (AD) were assessed during the EZM.

n = 8 (CD-AAV-mCherry), 9 (CD-AAV-hM3Dq), 8 (CD-AAV-hM4Di), 9 (ARS-CD-AAV-mCherry), 9 (ARS-CD-AAV-hM3Dq), 8 (ARS-CD-AAV-hM4Di), 9 (ARS-HFD-AAV-mCherry), 9 (ARS-HFD-AAV-hM3Dq), or 9 (ARS-HFD-AAV-hM4Di) mice per group.

The data in (B), (E) to (H), (L) to (M), (O) to (T), (V) to (Y), and (AA) to (AD) are presented as mean ± SEM.

*$P < 0.05$, **$P < 0.01$, #$P < 0.001$, P-values are calculated using one-way ANOVA with Tukey correction (B-J, L-T, V-AD).

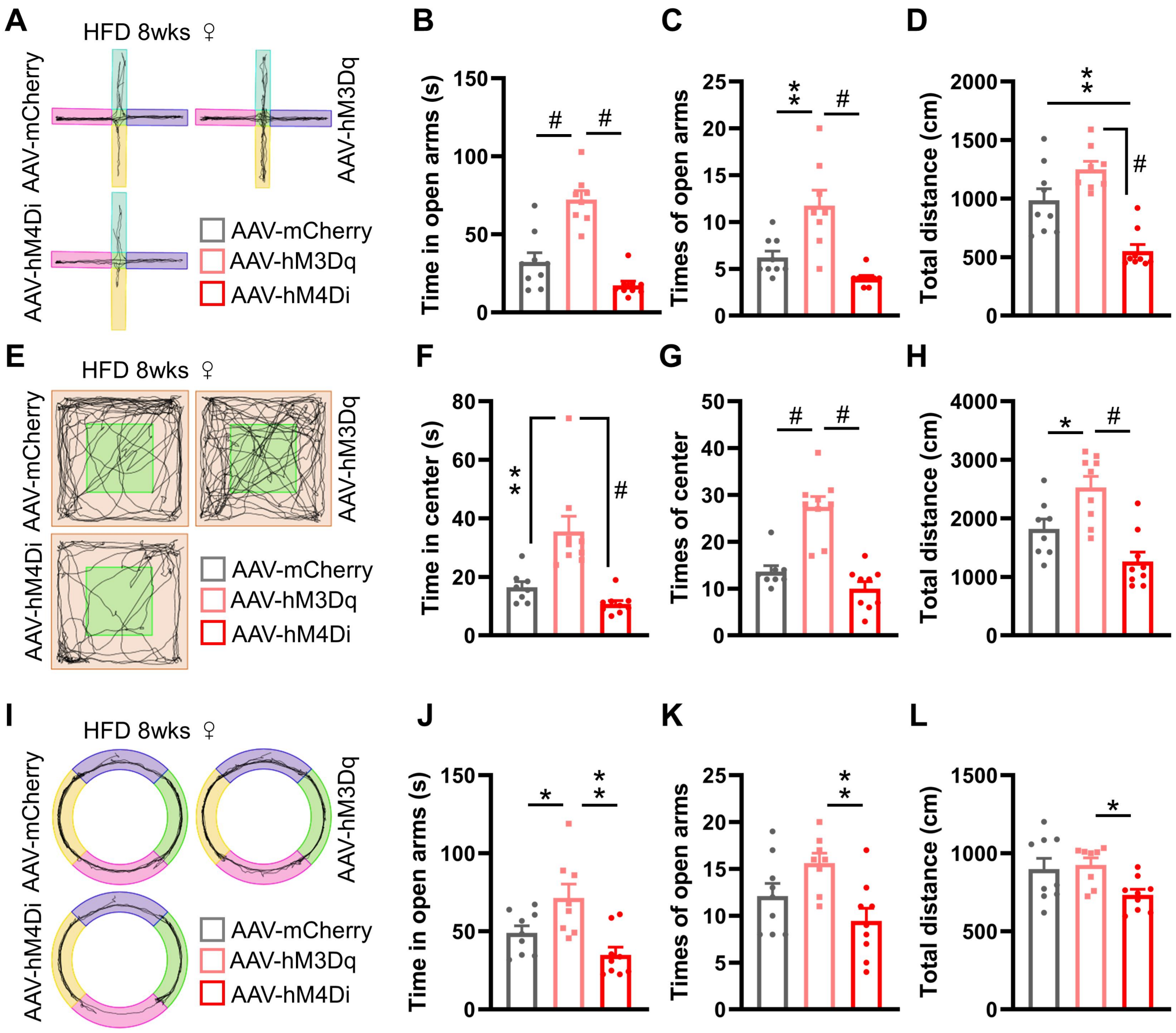

Figure S7. Activation of PVN Oxt neurons in female mice can effectively reverse anxiety-like behaviors induced by a long-term HFD.

(A–D) 8-week-old *Oxt*^{Cre} female mice were injected with Cre inducible AAV-mCherry, AAV-hM3Dq, and AAV-hM4Di viruses into the PVN and fed a HFD for 8 weeks. The activity trajectory line plot (A), time in open arms (B), times of open arms (C), and total distance (D) of the AAV-mCherry, AAV-hM3Dq, and AAV-hM4Di mice were assessed in the EPM. n = 9 (AAV-mCherry), 8 (AAV-hM3Dq), or 9 (AAV-hM4Di) mice per group.

(E–H) The mice activity trajectory line plot (E), time in center (F), times of center (G), and total distance (H) were assessed in the OFT. n = 8 (AAV-mCherry), 9 (AAV-hM3Dq), or 9 (AAV-hM4Di) mice per group.

(I–L) The mice activity trajectory line plot (I), time in open arms (J), times of open arms (K),

and total distance (L) were assessed during the EZM. n = 9 (AAV-mCherry), 8 (AAV-hM3Dq), or 9 (AAV-hM4Di) mice per group.

The data in (B) to (D), (F) to (H), and (J) to (L) are presented as mean ± SEM.

*P < 0.05, **P < 0.01, #P < 0.001, P-values are calculated using one-way ANOVA with Tukey correction (B-D, F-H, J-L).

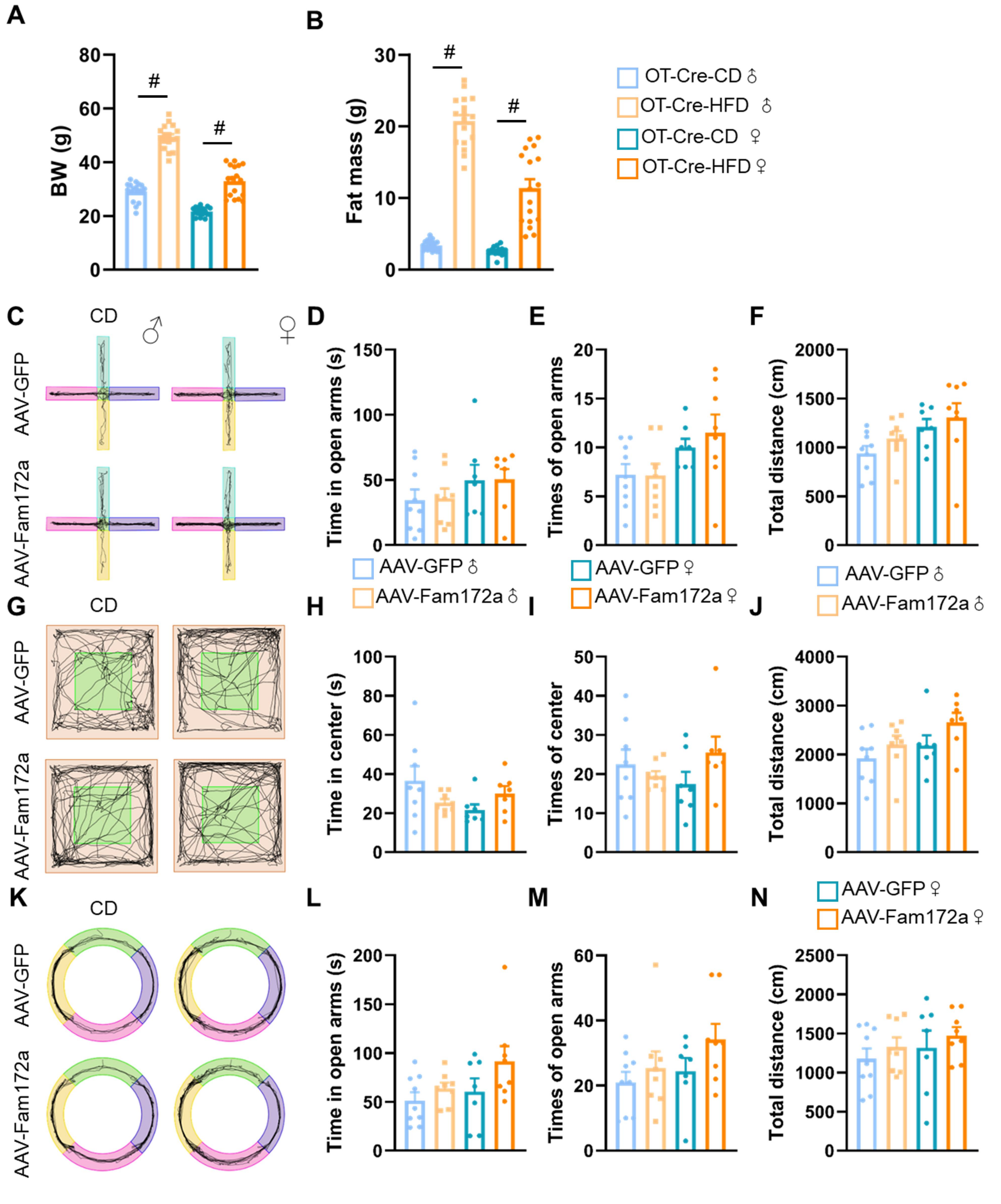

Figure S8. Overexpression of Fam172a in Oxt neurons within the PVN marginally influences on anxiety-related behaviors in CD mice.

(A and B) 8-week-old female Oxt^{Cre} mice were injected with either Cre-inducible AAV-GFP or AAV-Fam172a viruses into the PVN and subsequently fed a CD or a HFD for 8 weeks. Following the assessment, body weight (A) and body composition (B) were measured. n = 17

(OT-Cre-CD, male), 17 (OT-Cre-HFD, male), 16 (OT-Cre-CD, female), or 17 (OT-Cre-HFD, female) mice per group.

(C–F) The anxiety-like behavior of mice injected with AAV-GFP or AAV-Fam172a was assessed while they were fed a CD. The activity trajectory line plot (C), time in open arms (D), times of open arms (E), and total distance (F) of AAV-GFP or AAV-Fam172a mice were assessed in the EPM. n = 9 (AAV-GFP, male), 8 (AAV-Fam172a, male), 7 (AAV-GFP, female), or 8 (AAV-Fam172a, female) mice per group.

(G–J) The mice activity trajectory line plot (G), time in center (H), times of center (I), and total distance (J) were assessed in the OFT. n = 8 (AAV-GFP, male), 8 (AAV-Fam172a, male), 7 (AAV-GFP, female), or 7 (AAV-Fam172a, female) mice per group.

(K–N) The mice activity trajectory line plot (K), time in open arms (L), times of open arms (M), and total distance (N) were assessed during the EZM. n = 9 (AAV-GFP, male), 8 (AAV-Fam172a, male), 7 (AAV-GFP, female), or 8 (AAV-Fam172a, female) mice per group.

The data in (A) and (B) are presented as mean ± SEM.

#P < 0.001, P-values are calculated using two-way ANOVA with Tukey correction (A, B, D-F, H-J, L-N).

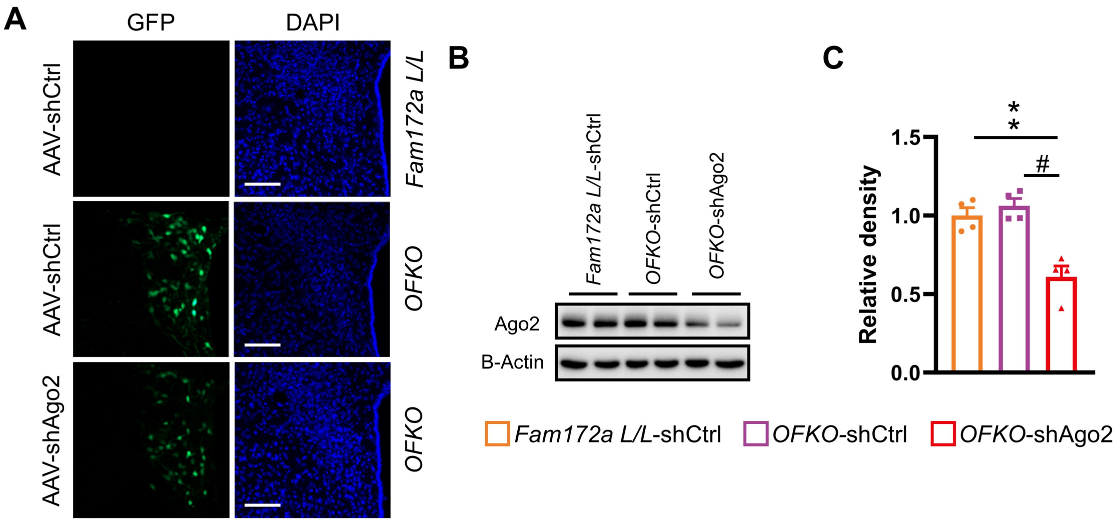

Figure S9. Establishment of the specific knockdown model targeting Ago2 in Oxt within the PVN mice.

(A) The AAV-FLEX-shAgo2-EGFP virus, designed to knockdown Ago2, was injected into the PVN of *OFKO* mice (*OFKO*-shAgo2). Meanwhile, a control virus, AAV-FLEX-shCtrl-EGFP, was administered to *Fam172a L/L* mice (*Fam172a L/L*-shCtrl) and another group of *OFKO* mice (*OFKO*-shCtrl), both targeting the same region. The Cre-dependent AAV-shCtrl and AAV-shAgo2, had the potential to induce the expression of EGFP, resulting in a green fluorescent signal. Cell nuclei were counter-stained with DAPI (blue). Scale bars, 100 μm.

(B and C) *Fam172a L/L*-shCtrl, *OFKO*-shCtrl, and *OFKO*-shAgo2 mice hypothalamic lysates were extracted for Western blot analysis of Ago2 (B), and quantification of the Western blots for *Fam172a L/L*-shCtrl, *OFKO*-shCtrl, and *OFKO*-shAgo2 mice (C). n = 4 for each group.

The data in (C) are presented as mean ± SEM.

**P < 0.01, #P < 0.001, P-values are calculated using one-way ANOVA with Tukey correction (C).

Table 1. Primer sequences

Gene	Primer	Sequence (5'->3')
CPLX1	Forward	CAGGGTATAAGAGACAAGTATGGCA
	Reverse	GGGATAGCCTTCTTGGGTCG
Fam172a	Forward	AGGACGAACCACCTTTTGATTT
	Reverse	CACCTGTGTAAGTCTTCTCGGTA
WDR19	Forward	GAAGCCTTCACCTTGGCTCA
	Reverse	AAGTGCTTTAGTGCCCGTGA
C11orf80	Forward	TTCTCAAGACGCATCAGGCA
	Reverse	TTCACCGCGACACTGAAAGA
DNASE2	Forward	TGGCAAGACGGTGTAGGGTA
	Reverse	TTAGCTGCTTGCCCTTCGTA
USP6NL	Forward	TGTGAGCGTGTGGCTGTATT
	Reverse	GATGCAGCTGACCCACTCTT
IQCC	Forward	AGGCCTATGAGAAGACCTCGT
	Reverse	TAGCAGAGCTCCCTGCCTTTA
RALGDS	Forward	CGAGGCTGAGGATGATGGTAG
	Reverse	ACTCGTTCTCACACCCTAGC

Table 2. Statistics

**Figure**	**Statistics**
Figure. 1B	*P*<0.0001 (CD♂ vs. HFD 8wks♂), *P*=0.0055 (CD♀ vs. HFD 8wks♀), *F*(3, 19)=135.8 for group.
Figure. 1C	*P*<0.0001 (CD♂ vs. HFD 8wks♂), *P*=0.0006 (CD♀ vs. HFD 8wks♀), *F*(3, 19)=89.11 for group.
Figure. 1D	*P*=0.0181 (CD♂ vs. HFD 8wks♂), *P*=0.0247 (CD♀ vs. HFD 8wks♀), *F*(3, 12)=8.040 for group.
Figure. 1F	*P*=0.0002 (CD♂ vs. HFD 8wks♂), *P*=0.0200 (CD♀ vs. HFD 8wks♀), *F*(3, 19)=13.41 for group.
Figure. 1G	*P*=0.0036 (CD♀ vs. HFD 8wks♀), *F*(3, 19)=7.488 for group.
Figure. 1H	*P*=0.0205 (CD♀ vs. HFD 8wks♀), *F*(3, 19)=4.074 for group.
Figure. 1J	*P*=0.0275 (CD♂ vs. HFD 8wks♂), *P*=0.0520 (CD♀ vs. HFD 8wks♀), *F*(3, 19)=6.074 for group.
Figure. 1N	*P*<0.0001 (CD♂ vs. HFD 8wks♂), *P*=0.0353 (CD♀ vs. HFD 8wksf♀), *F*(3, 19)=15.99 for group.
Figure. 1O	*P*=0.0002 (CD♂ vs. HFD 8wks♂), *P*=0.0014 (CD♀ vs. HFD 8wks♀), *F*(3, 19)=18.96 for group.
Figure. 1P	*P*<0.0001 (CD♀ vs. HFD 8wks♀), *F*(3, 19)=14.25 for group.
Figure. 2B	*P*<0.001 (AAV-mCherry♂ vs. AAV-hM3Dq♂), *P*=0.0042 (AAV-mCherry♂ vs. AAV-hM4Di♂), *P*<0.001 (AAV-hM3Dq♂ vs. AAV-hM4Di♂). *F*(2, 8)=323.5 for treatment.
Figure. 2E	*P*=0.037 (AAV-mCherry♂ vs. AAV-hM3Dq♂), *P*=0.0025 (AAV-hM3Dq♂ vs. AAV-hM4Di♂). *F*(2, 23)=7.530 for treatment.
Figure. 2F	*P*=0.0322 (AAV-hM3Dq♂ vs. AAV-hM4Di♂). *F*(2, 23)=4.424 for treatment.
Figure. 2G	*P*=0.0025 (AAV-mCherry♂ vs. AAV-hM4Di♂), *P*=0.0405 (AAV-hM3Dq♂ vs. AAV-hM4Di♂). *F*(2, 23)=7.641 for treatment.
Figure. 2I	*P*=0.0483 (AAV-mCherry♂ vs. AAV-hM3Dq♂), *P*=0.0215 (AAV-hM3Dq♂ vs. AAV-hM4Di♂). *F*(2, 23)=4.871 for treatment.
Figure. 2K	*P*=0.0348 (AAV-hM3Dq♂ vs. AAV-hM4Di♂). *F*(2, 23)=3.578 for treatment.

Figure. 2M	*P*=0.0351 (AAV-mCherry♂ vs. AAV- hM3Dq♂), *P*=0.0016 (AAV-hM3Dq♂ vs. AAV-hM4Di♂). *F*(2, 21)=8.480 for treatment.
Figure. 2N	*P*=0.0084 (AAV-hM3Dq♂ vs. AAV-hM4Di♂). *F*(2, 21)=5.574 for treatment.
Figure. 2O	*P*=0.0838 (AAV-mCherry♂ vs. AAV-hM3Dq♂), *P*=0.0483 (AAV-mCherry♂ vs. AAV-hM4Di♂), *P*<0.001 (AAV-hM3Dq♂ vs. AAV-hM4Di♂). *F*(2, 21)=10.56 for treatment.
Figure. 3B	*P*=0.0013, *t*(6)=5.636 (PSMB4), *P*=0.0021, *t*(6)=5.179 (Fam172a), *P*=0.0106, *t*(6)=3.656 (PEX11G), *P*=0.0134, *t*(6)=3.463 (KRTCAP3), *P*=0.0195, *t*(6)=3.161 (RIOK3), *P*=0.0297, *t*(6)=2.836 (C11ORF80), *P*=0.0272, *t*(6)=2.904 (DNASE2), *P*=0.0303, *t*(6)=2.821 (CD47), *P*=0.0349, *t*(6)=2.715 (CPLX1).
Figure. 3D	*P*=0.0238, *t*(4)=3.549.
Figure. 3F	*P*<0.0001, *t*(8)=7.904.
Figure. 4D	*P*=0.0010, *t*(4)=8.512.
Figure. 4E	*P*=0.0278 (AAV-GFP♂ vs. AAV-Fam172a♂), *P*=0.0412 (AAV-GFP♀ vs. AAV-Fam172a♀), *F*(5,13)=1.853 for gender, *F*(3, 13)=7.018 for group.
Figure. 4G	*P*=0.0422 (AAV-GFP♂ vs. AAV-Fam172a♂), *P*=0.0012 (AAV-GFP♀ vs. AAV-Fam172a♀), *F*(8,20)=1.626 for gender, *F*(3, 20)=9.774 for group.
Figure. 4K	*P*=0.0320 (AAV-GFP♂ vs. AAV-Fam172a♂), *F*(8,20)=1.836 for gender, *F*(3, 20)=4.186 for group.
Figure. 4L	*P*=0.0039 (AAV-GFP♂ vs. AAV-Fam172a♂), *F*(8,20)=2.042 for gender, *F*(3, 20)=9.782 for group.
Figure. 4O	*P*=0.0353 (AAV-GFP♂ vs. AAV-Fam172a♂), *P*=0.0018 (AAV-GFP♀ vs. AAV-Fam172a♀), *F*(7,18)=0.7962 for gender, *F*(3, 18)=13.09 for group.
Figure. 4P	*P*=0.0057 (AAV-GFP♂ vs. AAV-Fam172a♀), *P*=0.0016 (AAV-Fam172a♂ vs. AAV-Fam172a♀), *F*(7,18)=0.5075 for gender, *F*(3, 18)=8.009 for group.
Figure. 5B	*P*=0.0492 (*Fam172a L/L*♂ vs. *OFKO*♂), *P*=0.0088 (*Fam172a L/L*♀ vs. *OFKO*♀), *F*(5,13)=0.3222 for gender, *F*(3, 13)=8.312 for group.

Figure. 5D	*P*=0.0099 (*Fam172a L/L*♂ vs. *OFKO*♂), *P*=0.0282 (*Fam172a L/L*♀ vs. *OFKO*♀), *F*(9, 25)=0.8234 for gender, *F*(3, 25)=7.312 for group.
Figure. 5E	*P*=0.0067 (*Fam172a L/L*♂ vs. *OFKO*♂), *F*(11, 23)=0.6730 for gender, *F*(3, 23)=6.338 for group.
Figure. 5H	*P*=0.0028 (*Fam172a L/L*♂ vs. *OFKO*♂), *P*=0.0328 (*Fam172a L/L*♀ vs. *OFKO*♀), *F*(12, 25)=2.239 for gender, *F*(3, 25)=8.147 for group.
Figure. 5I	*P*=0.0306 (*OFKO*♂ vs. *OFKO*♀), *F*(12, 25)=0.9619 for gender, *F*(3, 25)=5.051 for group.
Figure. 5J	*P*=0.0088 (*Fam172a L/L*♂ vs. *OFKO*♂), *F*(12, 25)=0.8339 for gender, *F*(3, 25)=9.955 for group.
Figure. 5L	*P*=0.0246 (*Fam172a L/L*♂ vs. *OFKO*♂), *P*=0.0194 (*Fam172a L/L*♀ vs. *OFKO*♀), *F*(10, 25)=1.747 for gender, *F*(3, 25)=7.471 for group.
Figure. 5N	*P*=0.0039 (*Fam172a L/L*♀ vs. *OFKO*♀), *F*(10, 25)=2.112 for gender, *F*(3, 25)=11.79 for group.
Figure. 6C	*P*=0.0150, *t*(4)=4.085 (Fam-Total), *P*=0.0286, *t*(4)=3.350 (Fam-Cytoplasm), *P*=0.0357, *t*(4)=3.114 (Ago2-Cytoplasm), *P*=0.0386, *t*(4)=3.036 (Fam-Nucleus), *P*=0.0447, *t*(4)=2.886 (Ago2-Nucleus).
Figure. 6D	*P*=0.0174 (ADV-shCtrl, NaCl vs. ADV-shFam, NaCl), *P*=0.8228 (ADV-shCtrl, NaCl vs. ADV-shFam, BCI-137), *P*=0.0460 (ADV-shFam, NaCl vs. ADV-shFam, BCI-137), *F*(2, 11)=6.092 for treatment.
Figure. 6E	Time=90 min, *P*=0.0333 (ADV-shCtrl, NaCl vs. ADV-shFam, NaCl), *P*=0.3157 (ADV-shCtrl, NaCl vs. ADV-shFam, BCI-137), *P*=0.0192 (ADV-shFam, NaCl vs. ADV-shFam, BCI-137), *F*(2.063, 22.69)=60.15 for Time, *F*(2, 11)=12.72 for treatment.
Figure. 7B	*P*=0.0322 (*Fam172a L/L*-shCtrl♂ vs. *OFKO*-shCtrl♂), *P*=0.9537 (*Fam172a L/L*-shCtrl♂ vs. *OFKO*-shAgo2♂), *P*=0.0167 (*OFKO*-shCtrl♂ vs. *OFKO*-shAgo2♂), *F*(2, 24)=5.453 for treatment.
Figure. 7E	*P*=0.0038 (*Fam172a L/L*-shCtrl♀ vs. *OFKO*-shCtrl♀), *P*=0.5853 (*Fam172a L/L*-shCtrl♀ vs. *OFKO*-shAgo2♀), *P*=0.0393 (*OFKO*-shCtrl♀ vs. *OFKO*-shAgo2♀), *F*(2, 22)=7.207 for treatment.
Figure. 7F	*P*=0.0005 (*Fam172a L/L*-shCtrl♀ vs. *OFKO*-shCtrl♀), *F*(2, 22)=10.10 for treatment.

Figure. 7I	$P<0.001$ (*Fam172a L/L*, NaCl♂ vs. *OFKO*, NaCl♂), $P=0.1961$ (*Fam172a L/L*, NaCl♂ vs. *OFKO*, Oxt♂), $P=0.0404$ (*OFKO*, NaCl♂ vs. *OFKO*, Oxt♂), $F(2, 23)=9.480$ for treatment.
Figure. 7J	$P=0.0237$ (*Fam172a L/L*, NaCl♂ vs. *OFKO*, NaCl♂), $P=0.9958$ (*Fam172a L/L*, NaCl♂ vs. *OFKO*, Oxt♂), $P=0.0196$ (*OFKO*, NaCl♂ vs. *OFKO*, Oxt♂), $F(2, 23)=5.473$ for treatment.
Figure. 7K	$P=0.1030$ (*Fam172a L/L*, NaCl♂ vs. *OFKO*, NaCl♂), $P=0.7303$ (*Fam172a L/L*, NaCl♂ vs. *OFKO*, Oxt♂), $P=0.0221$ (*OFKO*, NaCl♂ vs. *OFKO*, Oxt♂), $F(2, 23)=4.418$ for treatment.
Figure. 7L	$P=0.0034$ (*Fam172a L/L*, NaCl♀ vs. *OFKO*, NaCl♀), $P=0.8422$ (*Fam172a L/L*, NaCl♀ vs. *OFKO*, Oxt♀), $P=0.0274$ (*OFKO*, NaCl♀ vs. *OFKO*, Oxt♀), $F(2, 27)=7.267$ for treatment.
Figure. 7M	$P=0.0132$ (*Fam172a L/L*, NaCl♀ vs. *OFKO*, NaCl♀), $P=0.8995$ (*Fam172a L/L*, NaCl♀ vs. *OFKO*, Oxt♀), $P=0.0628$ (*OFKO*, NaCl♀ vs. *OFKO*, Oxt♀), $F(2, 27)=5.284$ for treatment.
Figure. 7N	$P=0.0093$ (*Fam172a L/L*, NaCl♀ vs. *OFKO*, NaCl♀), $P=0.2068$ (*Fam172a L/L*, NaCl♀ vs. *OFKO*, Oxt♀), $P=0.4648$ (*OFKO*, NaCl♀ vs. *OFKO*, Oxt♀), $F(2, 27)=5.194$ for treatment.
Figure. S1B	$P=0.0074$, $t(17)=3.038$ (RS-1d), $P=0.0020$, $t(17)=3.647$ (RS-2d), $P=0.0009$, $t(17)=4.030$ (RS-3d), $P<0.0001$, $t(17)=5.276$ (RS-4d), $P=0.0272$, $t(17)=5.519$ (RS-5d), $P=0.0303$, $t(17)=5.358$ (RS-6d), $P=0.0349$, $t(17)=4.885$ (RS-7d).
Figure. S1D	$P=0.0294$, $t(19)=2.356$.
Figure. S1E	$P=0.0409$, $t(19)=2.193$.
Figure. S1F	$P=0.0026$, $t(19)=3.469$.
Figure. S1G	$P=0.0422$, $t(19)=2.179$.
Figure. S1I	$P=0.0239$, $t(15)=2.512$.
Figure. S1M	$P=0.0459$, $t(19)=2.137$.
Figure. S1N	$P=0.0459$, $t(19)=2.137$.
Figure. S1O	$P=0.0427$, $t(19)=2.172$.
Figure. S2B	$P=0.0058$ (CD♂ vs. ARS-HFD♂), $P=0.0211$ (ARS-CD♂ vs. ARS-HFD♂), $F(2, 24)=6.837$ for group.
Figure. S2C	$P=0.0385$ (CD♂ vs. ARS-CD♂), $P=0.0008$ (CD♂ vs. ARS-HFD♂),

	$F(2, 24)=9.103$ for group.
Figure. S2F	$P=0.0139$ (CD♂ vs. ARS-HFD♂), $F(2, 24)=4.997$ for group.
Figure. S2J	$P=0.0189$ (CD♂ vs. ARS-HFD♂), $F(2, 24)=4.347$ for group.
Figure. S2M	$P=0.0046$ (CD♂ vs. ARS-CD♂), $P=0.0004$ (ARS-CD♂ vs. ARS-HFD♂), $F(2, 12)=15.96$ for group.
Figure. S3B	$P=0.0472$ (CD♀ vs. ARS-HFD♀), $F(2, 23)=3.383$ for group.
Figure. S3C	$P=0.0043$ (CD♀ vs. ARS-HFD♀), $P=0.0090$ (ARS-CD♀ vs. ARS-HFD♀), $F(2, 23)=7.965$ for group.
Figure. S3D	$P=0.0320$ (ARS-CD♀ vs. ARS-HFD♀), $F(2, 23)=3.817$ for group.
Figure. S3F	$P=0.0279$ (CD♀ vs. ARS-HFD♀), $P=0.0442$ (ARS-CD♀ vs. ARS-HFD♀), $F(2, 21)=4.846$ for group.
Figure. S3G	$P=0.0375$ (CD♀ vs. ARS-HFD♀), $F(2, 21)=3.857$ for group.
Figure. S3J	$P=0.0255$ (CD♀ vs. ARS-HFD♀), $P=0.0388$ (ARS-CD♀ vs. ARS-HFD♀), $F(2, 23)=5.004$ for group.
Figure. S3K	$P=0.0453$ (CD♀ vs. ARS-HFD♀), $F(2, 23)=3.636$ for group.
Figure. S3M	$P=0.0182$ (CD♀ vs. ARS-CD♀), $P=0.0061$ (ARS-CD♀ vs. ARS-HFD♀), $F(2, 12)=8.529$ for group.
Figure. S4B	$P=0.0003$ (CD vs. ARS), $P<0.0001$ (ARS vs. CRS), $F(2, 12)=28.11$ for group.
Figure. S4D	$P=0.0008$ (CD vs. ARS-CD), $P=0.0015$ (ARS-CD vs. ARS-HFD), $F(2, 12)=15.72$ for group.
Figure. S4F	$P=0.0260$, $t(8)=2.726$.
Figure. S5B	$P=0.0271$ (AAV-mCherry♂ vs. AAV-hM4Di♂), $P=0.0133$ (AAV-hM3Dq♂ vs. AAV-hM4Di♂), $F(2, 31)=5.655$ for group.
Figure. S5C	$P=0.0010$ (AAV-mCherry♂ vs. AAV-hM4Di♂), $P<0.0001$ (AAV-hM3Dq♂ vs. AAV-hM4Di♂), $F(2, 31)=13.92$ for group.
Figure. S5D	$P=0.0226$ (AAV-mCherry♂ vs. AAV-hM4Di♂), $P=0.0019$ (AAV-hM3Dq♂ vs. AAV-hM4Di♂), $F(2, 31)=7.806$ for group.
Figure. S5E	$P=0.0160$ (AAV-mCherry♂ vs. AAV-hM4Di♂), $P=0.0111$ (AAV-hM3Dq♂ vs. AAV-hM4Di♂), $F(2, 31)=6.210$ for group.
Figure. S5F	$P=0.0125$ (AAV-hM3Dq♂ vs. AAV-hM4Di♂), $F(2, 31)=4.839$ for group.
Figure. S5H	$P=0.0392$ (AAV-mCherry♂ vs. AAV-hM3Dq♂), $P=0.0229$ (AAV-

	hM3Dq♂ vs. AAV-hM4Di♂), $F(2, 26)=4.868$ for group.
Figure. S5I	$P=0.0091$ (AAV-mCherry♂ vs. AAV-hM3Dq♂), $P=0.0135$ (AAV-hM3Dq♂ vs. AAV-hM4Di♂), $F(2, 26)=6.500$ for group.
Figure. S5J	$P=0.0082$ (AAV-mCherry♂ vs. AAV-hM3Dq♂), $F(2, 26)=5.425$ for group.
Figure. S5L	$P=0.0152$ (AAV-mCherry♂ vs. AAV-hM4Di♂), $P=0.0004$ (AAV-hM3Dq♂ vs. AAV-hM4Di♂), $F(2, 31)=10.04$ for group.
Figure. S5M	$P=0.0429$ (AAV-mCherry♂ vs. AAV-hM3Dq♂), $P=0.0010$ (AAV-hM3Dq♂ vs. AAV-hM4Di♂), $F(2, 31)=8.330$ for group.
Figure. S5N	$P=0.0386$ (AAV-hM3Dq♂ vs. AAV-hM4Di♂), $F(2, 31)=3.327$ for group.
Figure. S5O	$P=0.0002$ (AAV-hM3Dq♂ vs. AAV-hM4Di♂), $F(2, 31)=10.50$ for group.
Figure. S5P	$P=0.0002$ (AAV-hM3Dq♂ vs. AAV-hM4Di♂), $F(2, 31)=10.59$ for group.
Figure. S5Q	$P=0.0410$ (AAV-hM3Dq♂ vs. AAV-hM4Di♂), $F(2, 31)=3.627$ for group.
Figure. S5R	$P=0.0004$ (AAV-mCherry♂ vs. AAV-hM3Dq♂), $P=0.0004$ (AAV-hM3Dq♂ vs. AAV-hM4Di♂), $F(2, 27)=13.16$ for group.
Figure. S5S	$P=0.0105$ (AAV-mCherry♂ vs. AAV-hM3Dq♂), $P=0.0032$ (AAV-hM3Dq♂ vs. AAV-hM4Di♂), $F(2, 27)=7.788$ for group.
Figure. S5T	$P=0.0025$ (AAV-mCherry♂ vs. AAV-hM3Dq♂), $P=0.0081$ (AAV-hM3Dq♂ vs. AAV-hM4Di♂), $F(2, 27)=8.277$ for group.
Figure. S5V	$P=0.0080$ (AAV-mCherry♂ vs. AAV-hM4Di♂), $P<0.0001$ (AAV-hM3Dq♂ vs. AAV-hM4Di♂), $F(2, 31)=12.25$ for group.
Figure. S5W	$P=0.0165$ (AAV-mCherry♂ vs. AAV-hM4Di♂), $P=0.0055$ (AAV-hM3Dq♂ vs. AAV-hM4Di♂), $F(2, 31)=6.847$ for group.
Figure. S5X	$P=0.0186$ (AAV-mCherry♂ vs. AAV-hM4Di♂), $P=0.0018$ (AAV-hM3Dq♂ vs. AAV-hM4Di♂), $F(2, 31)=7.945$ for group.
Figure. S5Y	$P=0.0296$ (AAV-mCherry♂ vs. AAV-hM4Di♂), $P=0.0053$ (AAV-hM3Dq♂ vs. AAV-hM4Di♂), $F(2, 31)=6.502$ for group.
Figure. S5Z	$P=0.0262$ (AAV-mCherry♂ vs. AAV-hM4Di♂), $P=0.0003$ (AAV-hM3Dq♂ vs. AAV-hM4Di♂), $F(2, 31)=10.08$ for group.

Figure. S5AA	*P*=0.0062 (AAV-mCherry♂ vs. AAV-hM4Di♂), *P*=0.0009 (AAV-hM3Dq♂ vs. AAV-hM4Di♂), $F(2, 31)$=9.438 for group.
Figure. S5AB	*P*=0.0002 (AAV-mCherry♂ vs. AAV-hM3Dq♂), *P*=0.0002 (AAV-hM3Dq♂ vs. AAV-hM4Di♂), $F(2, 27)$=14.95 for group.
Figure. S5AC	*P*=0.0141 (AAV-mCherry♂ vs. AAV-hM3Dq♂), *P*=0.0146 (AAV-hM3Dq♂ vs. AAV-hM4Di♂), $F(2, 27)$=6.267 for group.
Figure. S5AD	*P*=0.0033 (AAV-mCherry♂ vs. AAV-hM3Dq♂), *P*=0.0003 (AAV-hM3Dq♂ vs. AAV-hM4Di♂), $F(2, 27)$=11.75 for group.
Figure. S6B	*P*=0.0109 (AAV-mCherry♀ vs. AAV-hM4Di♀), *P*=0.0433 (AAV-hM3Dq♀ vs. AAV-hM4Di♀), $F(2, 22)$=5.726 for group.
Figure. S6E	*P*=0.0302 (AAV-mCherry♀ vs. AAV-hM4Di♀), *P*=0.0453 (AAV-hM3Dq♀ vs. AAV-hM4Di♀), $F(2, 23)$=4.729 for group.
Figure. S6F	*P*=0.0093 (AAV-mCherry♀ vs. AAV-hM4Di♀), $F(2, 23)$=5.332 for group.
Figure. S6G	*P*=0.0165 (AAV-mCherry♀ vs. AAV-hM4Di♀), $F(2, 23)$=4.708 for group.
Figure. S6H	*P*=0.0039 (AAV-hM3Dq♀ vs. AAV-hM4Di♀), $F(2, 24)$=6.618 for group.
Figure. S6L	*P*=0.0024 (AAV-mCherry♀ vs. AAV-hM4Di♀), *P*=0.0409 (AAV-hM3Dq♀ vs. AAV-hM4Di♀), $F(2, 24)$=7.529 for group.
Figure. S6M	*P*=0.0143 (AAV-hM3Dq♀ vs. AAV-hM4Di♀), $F(2, 24)$=5.208 for group.
Figure. S6O	*P*=0.0383 (AAV-mCherry♀ vs. AAV-hM4Di♀), $F(2, 24)$=3.794 for group.
Figure. S6P	*P*=0.0389 (AAV-hM3Dq♀ vs. AAV-hM4Di♀), $F(2, 24)$=3.810 for group.
Figure. S6Q	*P*=0.0473 (AAV-mCherry♀ vs. AAV-hM4Di♀), $F(2, 24)$=3.310 for group.
Figure. S6R	*P*=0.0019 (AAV-hM3Dq♀ vs. AAV-hM4Di♀), $F(2, 24)$=7.668 for group.
Figure. S6S	*P*=0.0036 (AAV-hM3Dq♀ vs. AAV-hM4Di♀), $F(2, 24)$=6.759 for group.
Figure. S6T	*P*=0.0109 (AAV-hM3Dq♀ vs. AAV-hM4Di♀), $F(2, 24)$=5.084 for

	group.
Figure. S6V	*P*=0.0133 (AAV-mCherry♀ vs. AAV-hM4Di♀), *P*=0.0052 (AAV-hM3Dq♀ vs. AAV-hM4Di♀), *F*(2, 22)=7.383 for group.
Figure. S6W	*P*=0.0147 (AAV-hM3Dq♀ vs. AAV-hM4Di♀), *F*(2, 22)=5.117 for group.
Figure. S6X	*P*=0.0021 (AAV-mCherry♀ vs. AAV-hM4Di♀), *P*=0.0012 (AAV-hM3Dq♀ vs. AAV-hM4Di♀), *F*(2, 22)=10.67 for group.
Figure. S6Y	*P*=0.0208 (AAV-mCherry♀ vs. AAV-hM4Di♀), *P*=0.0027 (AAV-hM3Dq♀ vs. AAV-hM4Di♀), *F*(2, 23)=7.728 for group.
Figure. S6AA	*P*=0.0087 (AAV-mCherry♀ vs. AAV-hM4Di♀), *P*=0.0191 (AAV-hM3Dq♀ vs. AAV-hM4Di♀), *F*(2, 23)=6.421 for group.
Figure. S6AB	*P*=0.0057 (AAV-mCherry♀ vs. AAV-hM3Dq♀), *P*<0.0001 (AAV-hM3Dq♀ vs. AAV-hM4Di♀), *F*(2, 24)=16.02 for group.
Figure. S6AC	*P*=0.0118 (AAV-mCherry♀ vs. AAV-hM3Dq♀), *P*<0.0001 (AAV-hM3Dq♀ vs. AAV-hM4Di♀), *F*(2, 24)=15.17 for group.
Figure. S6AD	*P*=0.0084 (AAV-mCherry♀ vs. AAV-hM3Dq♀), *P*=0.0009 (AAV-hM3Dq♀ vs. AAV-hM4Di♀), *F*(2, 24)=9.833 for group.
Figure. S7B	*P*<0.0001 (AAV-mCherry♀ vs. AAV-hM3Dq♀), *P*<0.0001 (AAV-hM3Dq♀ vs. AAV-hM4Di♀), *F*(2, 23)=32.05 for group.
Figure. S7C	*P*=0.0017 (AAV-mCherry♀ vs. AAV-hM3Dq♀), *P*<0.0001 (AAV-hM3Dq♀ vs. AAV-hM4Di♀), *F*(2, 23)=16.20 for group.
Figure. S7D	*P*=0.0013 (AAV-mCherry♀ vs. AAV-hM4Di♀), *P*<0.0001 (AAV-hM3Dq♀ vs. AAV-hM4Di♀), *F*(2, 23)=21.06 for group.
Figure. S7F	*P*=0.0017 (AAV-mCherry♀ vs. AAV-hM3Dq♀), *P*<0.0001 (AAV-hM3Dq♀ vs. AAV-hM4Di♀), *F*(2, 23)=15.41 for group.
Figure. S7G	*P*<0.0001 (AAV-mCherry♀ vs. AAV-hM3Dq♀), *P*<0.0001 (AAV-hM3Dq♀ vs. AAV-hM4Di♀), *F*(2, 23)=28.81 for group.
Figure. S7H	*P*=0.0240 (AAV-mCherry♀ vs. AAV-hM3Dq♀), *P*<0.0001 (AAV-hM3Dq♀ vs. AAV-hM4Di♀), *F*(2, 23)=13.68 for group.
Figure. S7J	*P*=0.0483 (AAV-mCherry♀ vs. AAV-hM3Dq♀), *P*=0.0012 (AAV-hM3Dq♀ vs. AAV-hM4Di♀), *F*(2, 23)=8.569 for group.
Figure. S7K	*P*=0.0069 (AAV-hM3Dq♀ vs. AAV-hM4Di♀), *F*(2, 23)=5.737 for group.

Figure. S7L	*P*=0.0461 (AAV-hM3Dq♀ vs. AAV-hM4Di♀), *F*(2, 23)=3.922 for group.
Figure. S8A	*P*<0.0001 (OT-Cre-CD♂ vs. OT-Cre-HFD♂), *P*<0.0001 (OT-Cre-CD♀ vs. OT-Cre-HFD♀), *F*(3,45)=111.3 for gender.
Figure. S8B	*P*<0.0001 (OT-Cre-CD♂ vs. OT-Cre-HFD♂), *P*<0.0001 (OT-Cre-CD♀ vs. OT-Cre-HFD♀), *F*(3,45)=122.2 for gender.
Figure. S9C	*P*=0.0022 (*Fam172a L/L*-shCtrl vs. *OFKO*-shAgo2), *P*=0.0008 (*OFKO*-shCtrl vs. *OFKO*-shAgo2), *F*(2, 9)=18.96 for treatment.
